# New Electronic Transition of Ovalene in Solid *para*-H_2_: The S_1_–S_0_ Transition with Its Origin at 19 400 cm^–1^

**DOI:** 10.1021/acs.jpclett.5c03826

**Published:** 2026-01-30

**Authors:** Isabelle Weber, Johanna Langner, Henryk A. Witek, Yuan-Pern Lee

**Affiliations:** † Department of Applied Chemistry and Institute of Molecular Science, 34914 National Yang Ming Chiao Tung University, Hsinchu 3000093, Taiwan; ‡ Center for Emergent Functional Matter Science, 34914 National Yang Ming Chiao Tung University, Hsinchu 300093, Taiwan

## Abstract

As part of our ongoing efforts to evaluate solid *para*-H_2_ as a matrix host for electronic spectroscopy and to explore its potential in identifying carriers of the diffuse interstellar bands (DIBs), we previously reported the electronic spectrum of ovalene (C_32_H_14_), a medium-sized planar PAH with *D*_2*h*_ symmetry, in solid *para*-H_2_ and reassigned the previously reported S_1_–S_0_ spectral features to the S_2_–S_0_ transition with a lifetime of ∼1.7 μs ( WeberI., ,  J. Phys. Chem. Lett. 2024, 15, 10696 39413413 10.1021/acs.jpclett.4c02494PMC11514017). Extending our investigation to lower energies, we now report a second set of spectral features, previously unobserved, with a prominent origin band at ∼19 400 cm^–1^ in solid *para*-H_2_, approximately 1650 cm^–1^ below the previously reported S_2_–S_0_ origin at 21 050 cm^–1^, and a short fluorescence lifetime of ∼10 ns. We assigned these new features to the S_1_–S_0_ transition based on quantum-chemical calculations at the TD-B3LYP-D3BJ/6-311++G­(2d,2p) level and Franck–Condon Herzberg–Teller simulations.

## Graphical abstract

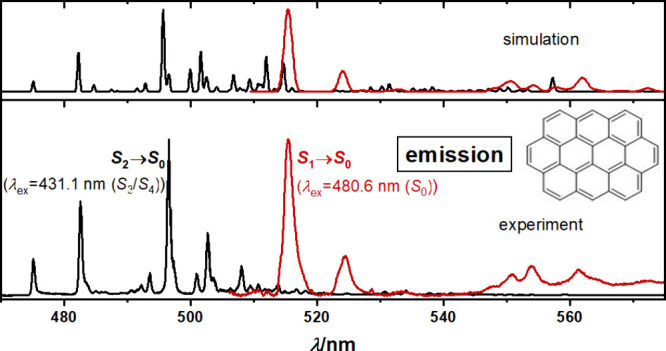

Over the past few decades, the planar *peri*-condensed polycyclic aromatic hydrocarbon (PAH) ovalene (C_32_H_14_) and its derivatives have garnered a significant amount of attention. They have been studied as intermediates in soot formation, as models for graphene and graphene-like systems in quantum-chemical investigations, as prospective functional materials, and as potential carriers of diffuse interstellar bands (DIBs). The DIBs are electronic or vibronic absorption bands of interstellar origin, spanning the ultraviolet (UV) to near-infrared (NIR) spectral range, with the highest density observed between 500 and 700 nm.^1–3^ First discovered by Heger in 1922,^4^ more than 500 DIBs have been catalogued to date. Despite extensive efforts, their molecular origins remain largely elusive. Only the buckminsterfullerene cation (C_60_^+^) has been successfully identified as the carrier of five DIBs at 934.91, 936.59, 942.58, 957.75, and 963.27 nm through comparison of spectra recorded using cryogenic ion-trap and Ne matrix isolation spectroscopy with astronomical observations.^5–8^

Large PAHs, particularly their cationic and protonated derivatives, are considered to be promising candidates for DIB carriers. However, their low vapor pressures pose challenges for obtaining laboratory spectra of these molecules under conditions comparable to the interstellar medium (ISM), namely, low temperatures and gas-phase environments. Matrix isolation techniques offer a viable alternative to commonly employed jet-expansion and cryogenic ion-trap methods, as they can isolate unstable species and require only minimal sample quantities. Nevertheless, spectra obtained via matrix isolation using noble gases often exhibit significant and unpredictable shifts in line positions relative to the gas-phase values due to distinct guest–host interactions. Compared with solid noble gases, the quantum solid *para*-hydrogen (*para*-H_2_) is soft and interacts only weakly with embedded guest molecules. In vibrational spectroscopy, matrix shifts in *para*-H_2_ relative to the gas phase are typically much less than 1%.^9,10^ For electronic spectra, our investigations of dispersed fluorescence and fluorescence excitation spectra of PAH containing fewer than 42 carbon atoms^11–14^ reveal a consistent red-shift of spectral lines relative to the gas phase, generally <110 cm^–1^, with an average of 70 ± 28 cm^–1^.^10^ Furthermore, protonated and hydrogenated molecules can be conveniently generated in solid *para*-H_2_ by electron bombardment of a mixture of the target molecule and *para*-H_2_ during deposition. This approach has enabled *para*-H_2_ matrix isolation spectroscopy to be employed for recording infrared (IR) and dispersed fluorescence and/or fluorescence excitation spectra of these astrochemically relevant molecules.^13,15–17^

As part of our efforts to evaluate *para*-H_2_ as a matrix host for electronic spectroscopy, we recorded the dispersed fluorescence and fluorescence excitation spectra of ovalene (C_32_H_14_).^14^ The fluorescence excitation spectrum consists of a weak origin band at 21 050 ± 3 cm^–1^ in solid *para*-H_2_, accompanied by vibronic transitions at 315, 865, and 1115 cm^–1^ to higher energies. This spectral pattern closely resembles the S_1_(*B*_2*u*_)–S_0_(*A_g_*) fluorescence excitation spectrum of jet-cooled C_32_H_14_ reported by Amirav et al.^18,19^ However, a peak-by-peak comparison of the two spectra suggested that the origin band reported by Amirav et al.^18,19^ at 21 449 ± 10 cm^–1^ should instead be assigned to the superposition of two vibronic transitions involving ν_45_ (*b*_1*g*_, 314 cm^–1^) and ν_23_ (*a_g_*, 319 cm^–1^). Consequently, the “true” origin band in the gas phase should be located at ∼21 130 cm^–1^. Further comparison of the observed dispersed fluorescence and fluorescence excitation spectra of C_32_H_14_ isolated in solid *para*-H_2_ with emission and absorption spectra simulated by a Franck–Condon Herzberg–Teller approach, using optimized geometries and scaled harmonic vibrational wavenumbers from (time-dependent) density functional theory ((TD-)­DFT) calculations, indicated that the observed spectra should in fact be reassigned to the S_2_(*B*_3*u*_)–S_0_(*A*_*g*_) transition rather than S_1_(*B*_2*u*_)–S_0_(*A*_*g*_) transition. This reassignment is supported by the relatively long radiative lifetimes observed, ∼1.7 μs in solid *para*-H_2_ and 1.7–2.4 μs in a supersonic jet,^18,19^ which are consistent with the predicted small oscillator strength of the S_2_(*B*_3*u*_) state (see Table 1); the agreement of fluorescence lifetimes of jet-cooled C_32_H_14_ and C_32_H_14_ isolated in solid *para*-H_2_ is consistent with our earlier experiments, e.g., on coronene (C_24_H_12_, τ_fl_^pH_2_^ ∼ 450 ns, τ_fl_^jet^ = 354–550 ns),^20,21^ suggesting a weak influence of the solid *para*-H_2_ environment on fluorescence lifetimes. In contrast, TD-DFT calculations for the S_1_(*B*_2*u*_) state yield an oscillator strength of 0.18, implying a significantly shorter fluorescence lifetime on the order of nanoseconds. Consistent with our proposed reassignment, we will refer to the spectra published by Amirav et al.^18,19^ as the S_2_(*B*_3*u*_)–S_0_(*A_g_*) spectra of jet-cooled C_32_H_14_, rather than the S_1_(*B*_2*u*_)–S_0_(*A*_*g*_) spectra, as in the original publications from 1980 and 1981.

**Table 1:** Vertical Transitions to the Six Lowest Electronic Excited Singlet States of C_32_H_14_ Predicted at the TD-B3LYP-G3BJ/6-311++G­(2d,2p) Level

state	symmetry	Δ*E*_vert_^abs^ (cm^–1^)	*f* ^a^	τ^b^ (ns)
S_1_	*B*_2*u*_	20 124	0.18	15
S_2_	*B*_3*u*_	21 769	∼0.00	1320
S_3_	*B*_1*g*_	25 294	0.00	230
S_4_	*B*_1*g*_	26 525	0.00	63
S_5_	*A*_*g*_	27 810	0.00	32
S_6_	*B*_3*u*_	28 610	1.05	1

a Oscillator strength.

b Radiative lifetimes estimated from the predicted excited-state properties as described in the Supporting Information of ref 14.

The properties of vertical transitions from the electronic ground state to the six lowest excited singlet states of C_32_H_14_, as predicted by TD-B3LYP-G3BJ/6-311++G­(2d,2p) calculations, are summarized in Table 1. Benkyi et al.^22^ previously demonstrated that TD-DFT failed to predict the correct order of lowest excited singlet states S_1_ and S_2_ for naphthalene and pyrene, two molecules also belonging to the *D*_2*h*_ point group. To the best of our knowledge, only one higher-level quantum-chemical investigation of the electronically excited states of C_32_H_14_ has been published to date. Employing the PPP-MRSDCI method, Ayanpour et al.^23^ predict the two lowest excited singlet one-photon states to be of *B*_2*u*_(S_1_) and *B*_3*u*_(S_2_) symmetry, respectively, consistent with our TD-DFT results. Although both S_2_(*B*_3*u*_) and S_6_(*B*_3*u*_) share the same symmetry, the S_2_(*B*_3*u*_)–S_0_(*A_g_*) transition exhibits an extremely small transition dipole moment, resulting in a near-zero oscillator strength (∼0.00). In contrast to the S*_n_* (*n* = 3–5)–S_0_(*A*_*g*_) transitions, which are symmetry forbidden, the transition between S_0_(*A_g_*) and S_2_(*B*_3*u*_) is symmetry allowed and the simulated emission and absorption spectra for this transition exhibit a weak 0_0_^0^ band. Simulated absorption and emission spectra for the three lowest-energy transitions, S_*n*_(*n* = 1–3)–S_0_, obtained by using a Franck–Condon Herzberg–Teller approach, are depicted in Figure S1. Absorption and emission spectra of the S_1_(*B*_2*u*_) and S_2_(*B*_3*u*_) states of C_32_H_14_ consist exclusively of vibrational normal modes of *a*_*g*_ and *b*_1*g*_ symmetry, while the absorption and emission spectra associated with the S_3_(*B*_1*g*_)–S_0_(*A_g_*) transition comprise vibrational normal modes of *b*_2*u*_ and *b*_3*u*_ symmetry. Consistent with the predicted oscillator strength, predicted relative intensities of the origin bands decrease in the following order: S_1_(*B*_2*u*_, *f* = 0.18, relative intensity (rel. int.) 1.0) > S_2_(*B*_3*u*_, *f* ∼ 0.00, rel. int. 0.2) > S_3_(*B*_1*g*_, *f* = 0.00, rel. int. 0.0, symmetry forbidden).

Our assignment of the dispersed fluorescence and fluorescence excitation spectra of C_32_H_14_ isolated in solid *para*-H_2_ reported in ref 14 to the S_2_(*B*_3*u*_)–S_0_(*A*_*g*_) transition and the subsequent reassignment of previously published literature results^18,19^ prompted a search for the “missing” S_1_(*B*_2*u*_) state. According to TD-DFT calculations, this state is expected to lie ∼1600 cm^–1^ lower in energy than the S_2_(*B*_3*u*_) state at 21 050 cm^–1^ in solid *para*-H_2_. To investigate this, we extended our excitation and detection ranges to longer wavelengths and observed an additional set of emission bands. This second emission system, depicted in Figure 1a, consists of a prominent feature at 19 400 ± 8 cm^–1^ in solid *para*-H_2_, ∼1650 cm^–1^ below the previously reported S_2_(*B*_3*u*_) origin band, and several significantly weaker bands at 333 (rel. int. ∼24%), 1246 (∼13%), 1350 (∼18%), and 1589 cm^–1^ (∼17%) to lower energies.

**Figure 1:**
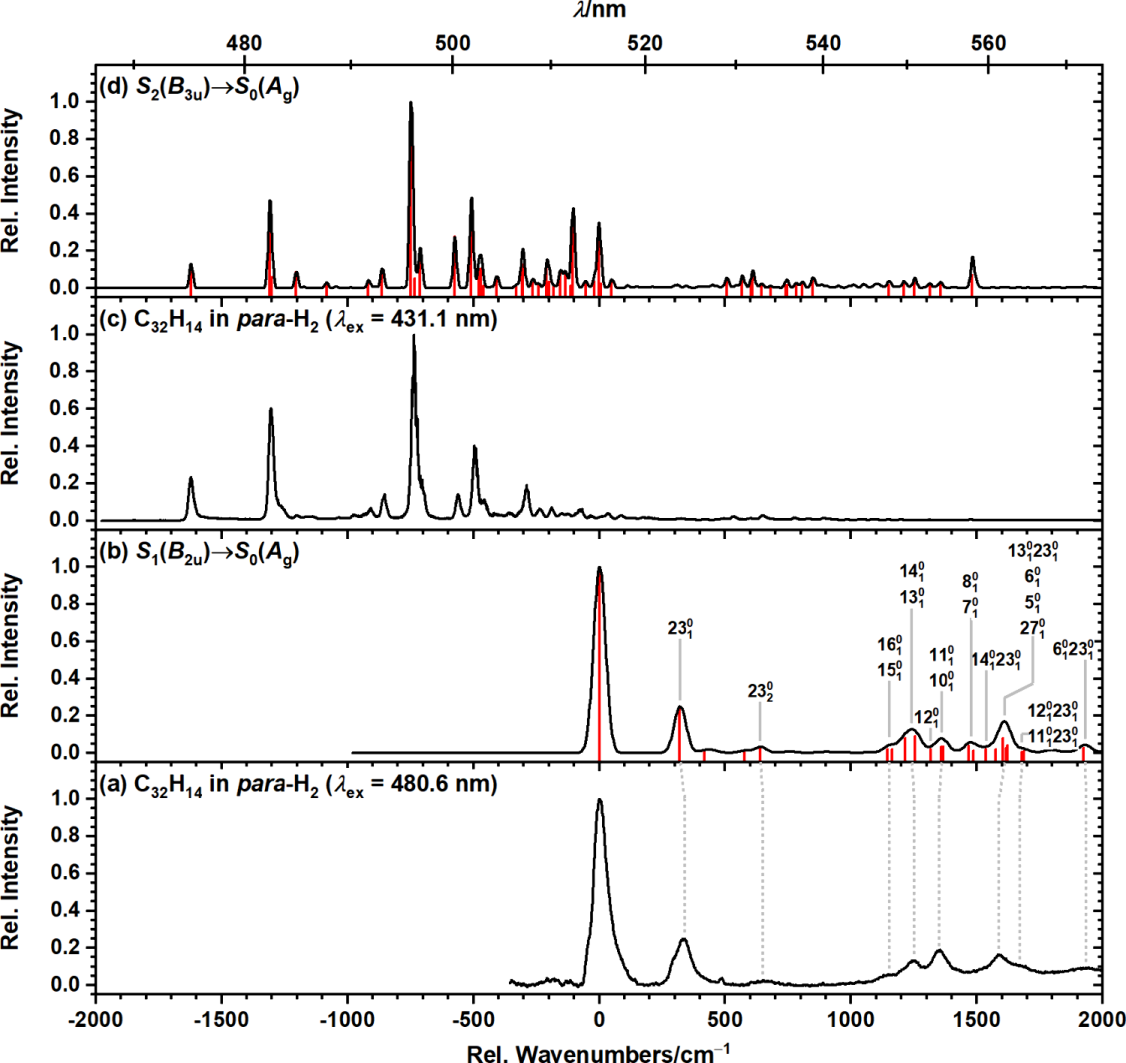
Comparison of dispersed fluorescence spectra from the two lowest electronic excited states of C_32_H_14_ isolated in solid *para*-H_2_. (a) Lower-energy emission recorded upon excitation of C_32_H_14_ isolated in solid *para*-H_2_ at 480.6 nm (20 807 cm^–1^). (b) Simulated S_1_ → S_0_ emission spectrum. (c) Previously reported emission spectrum recorded upon excitation at 431.1 nm (22 660 cm^–1^). (d) Simulated S_2_ → S_0_ emission spectrum. The stick spectra (red) were computed at the B3LYP-GD3BJ/6-311++G­(2d,2p) level of theory and convolved with a Gaussian line shape with full-widths at half-maximum (fwhm) of 55 cm^–1^ (for panel b) and 20 cm^–1^ (for panel d). Calculated harmonic vibrational wavenumbers were scaled by 0.98. The positions in wavenumbers (inverse centimeters) relative to the S_1_–S_0_ origin band are shown on the bottom axes.

Time-resolved fluorescence emission measurements in the range of 17 636–17 953 cm^–1^ (557.0–567.0 nm), following excitation at 19 444 cm^–1^ (514.3 nm), revealed an emission lifetime of 10 ± 1 ns for this band structure, as shown in Figure S2. This emission lifetime is significantly shorter than that of the S_2_–S_0_ transition of C_32_H_14_ isolated in solid *para*-H_2_ (1700 ± 10 ns)^14^ yet aligns with the lifetime of 15 ns estimated from our quantum-chemical calculations for a symmetry-allowed electronic transition with substantial oscillator strength (Table 1). The significantly different emission lifetimes, however, do not explain the notable differences in line width between the experimental spectra (cf. panels a and c of Figure 1), as the lifetime broadening can be estimated to be ∼0.0005 cm^–1^ for the shorter-lived S_1_(*B*_2*u*_) state; instead, line broadening will be mainly related to the coupling of vibronic energy levels of the isolated guest molecules to lattice vibrations of the *para*-H_2_ crystal. Comparison of the experimental dispersed fluorescence spectrum with simulated S_2_(*B*_3*u*_) → S_0_(*A*_*g*_) and S_1_(*B*_2*u*_) → S_0_(*A_g_*) emission spectra, depicted in Figure 1, strongly supports the assignment of the observed emission bands to fluorescence emission from the S_1_(*B*_2*u*_) state of C_32_H_14_. A peak-by-peak comparison between the experimental and simulated S_1_ → S_0_ emission spectra, along with assignments to vibrational normal modes, is provided in Table 2; corresponding band assignments are also indicated in Figure 1b. Although the mean absolute deviation in peak positions of 11 ± 7 cm^–1^ is slightly larger than in our previous studies of neutral PAH in solid *para*-H_2_,^11,13^ likely because of the significant overlap of vibronic contributions >1000 cm^–1^, it remains comparable to the mean absolute deviation in peak positions observed in our previous analysis of the S_2_(*B*_3*u*_) dispersed fluorescence spectrum of C_32_H_14_ in solid *para*-H_2_.^14^ A comparison of the present data with the IR spectrum of C_32_H_14_ in solid *para*-H_2_, reported by Tsuge et al.,^24^ is not feasible because of pronounced line broadening in the dispersed fluorescence spectrum and the different selection rules governing IR and vibronic transitions of S_1_(*Β*_2*u*_)–S_0_(*A_g_*), as dictated by group theory.

**Table 2:** Assignments for Bands Observed in the Dispersed Fluorescence Spectrum of C_32_H_14_ Isolated in Solid *para*-H_2_ and Comparison with the Peak Positions in the Simulated S_1_ → S_0_ Emission Spectrum

*para*-H_2_	B3LYP-GD3BJ				
LIF^a^ (cm^–1^)	FCHT^b^ (cm^–1^)	int.^c^ (%)	scaled^d^ (cm^–1^)	assignment	symmetry
0	0	100.0	0	0_0_^0^	*a*_*g*_
333	322	24.2	320	23_1_^0^	*a*_*g*_
657	640	3.0	640	23_2_^0^	*a*_*g*_
1154 (s)^e^	1154 (s)^e^	2.0	1144	16_1_^0^	*a*_*g*_
		2.3	1163	15_1_^0^	*a*_*g*_
1246	1243	8.2	1215	14_1_^0^	*a*_*g*_
		9.2	1255	13_1_^0^	*a*_*g*_
		2.3	1315	12_1_^0^	*a*_*g*_
1350	1360	3.5	1357	11_1_^0^	*a*_*g*_
		3.6	1366	10_1_^0^	*a*_*g*_
	(1478)	3.9	1468	8_1_^0^	*a*_*g*_
		1.5	1487	7_1_^0^	*a*_*g*_
		2.0	(1535)	14_1_^0^23_1_^0^	*a*_*g*_
1589	1610	2.3	(1575)	13_1_^0^23_1_^0^	*a*_*g*_
		8.2	1604	6_1_^0^	*a*_*g*_
		3.0	1617	5_1_^0^	*a*_*g*_
		4.1	1621	27_1_^0^	*b*_1*g*_
1671 (s)^e^	1681 (s)^e^	0.9	(1677)	12_1_^0^23_1_^0^	*a*_*g*_
		0.8	(1686)	11_1_^0^23_1_^0^	*a*_*g*_
1943	1931	2.9	(1924)	6_1_^0^23_1_^0^	*a*_*g*_

a Peak positions relative to the origin band at 19 400 cm^–1^.

b Peak positions relative to the origin band inferred from the computed stick spectrum after convolution with a Gaussian line shape with a fwhm of 55 cm^–1^. Features in parentheses were not observed experimentally.

c Relative percentage intensity referenced to the most intense band, the vibrationless transition. Only vibrational normal modes with a relative intensity ≥1.5% are listed.

d Harmonic vibrational wavenumbers scaled by 0.98. Values for overtone and combination bands correspond to the sum of the fundamental wavenumbers and are shown in parentheses.

e Shoulder.

By probing fluorescence emission in the range of 17 908–18 235 cm^–1^ (558.4–566.4 nm), corresponding to the overlapping low-intensity bands in the range of ∼1450–1700 cm^–1^ from the origin band in the dispersed fluorescence spectrum, and stepping the excitation wavelength in increments of 0.1 nm, we obtained the fluorescence excitation spectrum associated with the 19 400 cm^–1^ emission system, as depicted in Figure 2a. Similar to the corresponding dispersed fluorescence spectrum, the fluorescence excitation spectrum features an intense first band, likely the 0_0_^0^ band, followed by several weaker bands at 300 (rel. int. ∼60%), 631 (∼20%), 1217 (∼15%), 1337 (∼40%), 1557 (∼35%), and 1895 cm^–1^ (∼17%) to higher energies. Comparison of the experimental excitation spectrum with the simulated absorption spectrum for the S_1_(*B*_2*u*_) state of C_32_H_14_ in Figure 2 supports our assignment of the observed band system to the S_1_(*B*_2*u*_) ← S_0_(*A_g_*) transition. The agreement between experimental excitation and simulated absorption spectra is less satisfactory than that for the emission spectra. Vibrational band intensities in the experimental spectrum are about a factor of 2 higher, and the mean absolute deviation in peak positions, 23 ± 17 cm^–1^, is larger than in our previous studies of a PAH isolated in solid *para*-H_2_,^11,13,14^ likely due to uncertainties in the experimental peak positions due to significant line broadening and the somewhat low S/N ratio. The larger deviations in peak positions for the fluorescence excitation spectrum, as compared to the dispersed fluorescence spectrum, however, are consistent with our previous work^11,13,17^ and likely associated with the differences in the accuracy of (TD)-DFT for the description of the ground- and excited-state potential energy surfaces and the questionable applicability of ground-state vibrational scaling factors to excited-state vibrational wavenumbers. Detailed vibrational assignments are provided in Table 3 and are indicated in Figure 2b. The largest deviations in peak positions occur above 1200 cm^–1^, where most bands correspond to superpositions of several vibrational modes. Only contributions with relative intensities of ≥1.5% are included in Table 3 and Figure 2b; consequently, uncertainties in peak positions are greater in this spectral region.

**Figure 2:**
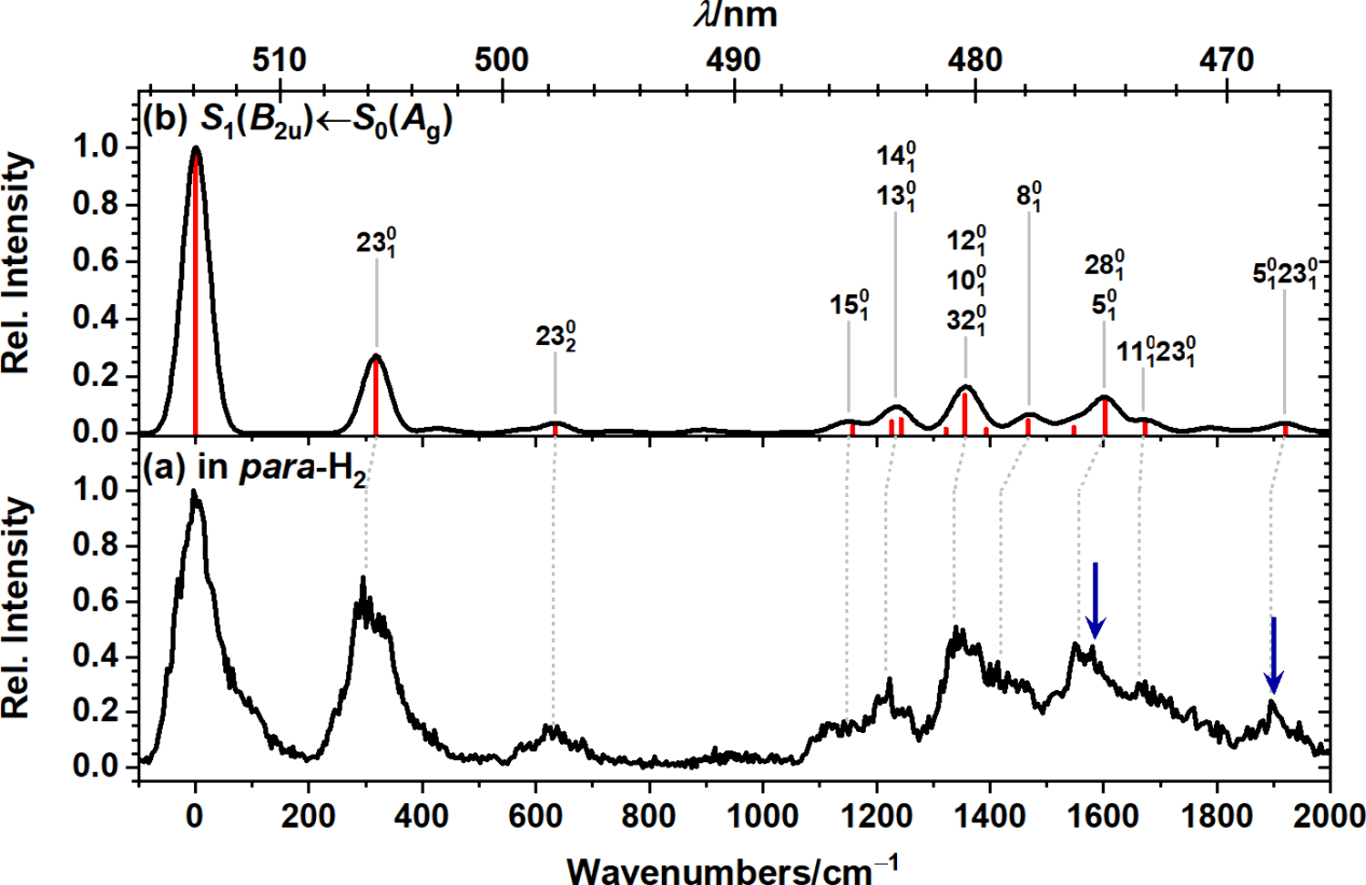
Comparison of the fluorescence excitation spectrum of C_32_H_14_ isolated in solid *para*-H_2_ with the simulated absorption spectrum of the S_1_ ← S_0_ transition. (a) Experimental spectrum obtained on probing emission in the range of 17 908–18 235 cm^–1^. (b) Simulated absorption spectrum of the S_1_ ← S_0_ transition. The computed stick spectrum (red) was convoluted with a Gaussian line shape with a fwhm of 55 cm^–1^ and shifted to align the origin bands. Harmonic vibrational wavenumbers were scaled by 0.98. Positions of peaks in the S_2_ fluorescence excitation spectrum of C_32_H_14_ in solid *para*-H_2_ reported in ref 14 are indicated by blue arrows.

**Table 3:** Assignments for Bands Observed in the Fluorescence Excitation Spectrum of C_32_H_14_ Isolated in Solid *para*-H_2_ and Comparison to Peak Positions in the Simulated S_1_ ← S_0_ Absorption Spectrum

*para*-H_2_	B3LYP-GD3BJ				
LIF^a^ (cm^–1^)	FCHT^b^ (cm^–1^)	int.^c^ (%)	scaled^d^ (cm^–1^)	assignment	symmetry
300	319	27.4	317	23_1_^0^	*a*_*g*_
631	634	3.6	634	23_2_^0^	*a*_*g*_
1147	1151	2.3	1157	15_1_^0^	*a*_*g*_
1217	1234	4.2	1227	14_1_^0^	*a*_*g*_
		5.0	1244	13_1_^0^	*a*_*g*_
1337	1357	1.6	1322	12_1_^0^	*a*_*g*_
		13.2	1356	11_1_^0^	*a*_*g*_
		1.5	1393	32_1_^0^	*b*_1*g*_
1419	1469	4.7	1467	8_1_^0^	*a*_*g*_
1557	1601	2.0	1547	28_1_^0^	*b*_1*g*_
		11.2	1603	5_1_^0^	*a*_*g*_
1663	1669	3.6	(1673)	11_1_^0^23_1_^0^	*a*_*g*_
1895	1919	3.0	(1919)	5_1_^0^23_1_^0^	*a*_*g*_

a Peak position relative to the origin band at 19 400 cm^–1^.

b Peak positions relative to the origin band inferred from the computed stick spectrum after convolution with a Gaussian line shape with a fwhm of 55 cm^–1^.

c Relative percentage intensity referenced to the most intense band, the vibrationless transition. Only vibrational normal modes with a relative intensity of ≥1.5% are listed.

d Harmonic vibrational wavenumbers scaled by 0.98. Values for overtone and combination bands corresponding to the sum of the fundamental wavenumbers are shown in parentheses.

In short, the observation of an additional red-shifted emission/absorption system originating at 19 400 ± 8 cm^–1^ in solid *para*-H_2_ under identical experimental conditions reinforces our previous reassignment of the gaseous spectrum of the S_1_(*B*_2*u*_)–S_0_(*A_g_*) transition to the S_2_(*B*_3*u*_)–S_0_(*A*_*g*_) transition of C_32_H_14_, with an origin band at 21 050 ± 3 cm^–1^ in solid *para*-H_2_. We attribute this newly observed system to the previously unreported S_1_(*B*_2*u*_) state of C_32_H_14_.

Emission from higher excited states, commonly termed anomalous fluorescence or anti-Kasha fluorescence,^25^ has been reported for a wide range of molecules, including azulenes,^26–28^ polyenes,^29–31^ thioketones,^32–34^ and several PAHs.^35–39^ In general, this phenomenon arises from either (i) inhibition of internal conversion from a higher to lower electronically excited state—due to a large energy gap, as in azulenes, or a scarcity of vibrational states that facilitate coupling of the electronically excited states near the potential energy surface minimum of the higher excited state, as in iso-quinoline—or (ii) repopulation of a strongly emitting higher excited state, for example, via thermal excitation, as observed in several PAHs.

Figure S3 depicts an extension of the excitation scan shown in Figure 2, covering higher excitation energies. In addition to the characteristic spectral signature assigned to the S_1_(*B*_2*u*_) ← S_0_(*A*_*g*_) transition spanning the range of 19 400–21 500 cm^–1^ in solid *para*-H_2_, the spectrum displays several weak but sharp features between 21 800 and 22 850 cm^–1^, along with two broad, intense features centered at ∼23 000 and ∼23 300 cm^–1^. Comparison with the previously reported fluorescence excitation spectrum obtained by probing emission from the S_2_ state^14^ clearly indicates that these features correspond to transitions previously assigned to S_2_(*B*_3*u*_) and S_3_(*B_1__g_*)/S_4_(*B_1g_*). Since the extended excitation spectrum was recorded by probing emission from S_1_(*B*_2*u*_), the results seem to suggest that excitation to a higher excited singlet state leads to the population of S_1_ via internal conversion. However, on close examination, although the selected probing region is far from the S_2_–S_0_ 0_0_^0^ band, weak features of the S_2_–S_0_ dispersed fluorescence spectrum extend into this range, as shown in Figure 1. This indicates that emission from S_1_ and S_2_ states was actually probed in this spectral region. Probing the emission from S_1_ at even longer wavelengths to avoid interference from S_2_ emission is experimentally infeasible. To clarify this, partially dispersed fluorescence spectra recorded at excitation wavelengths corresponding to states S_1_, S_2_, and S_3_/S_4_ are shown in Figure S4. The spectral pattern upon excitation at 514.1 nm (19 451 cm^–1^), near the origin band of the S_1_(*B*_2*u*_) ← S_0_(*A*_*g*_) excitation spectrum, is distinct from those recorded upon excitation at 456.3 nm (21 915 cm^–1^, S_2_) or 434.3 nm (23 026 cm^–1^, S_3_/S_4_), which match the previously reported S_2_(*B*_3*u*_) → S_0_(*A_g_*) emission. Upon excitation to higher excited singlet states S_3_(*B*_1*g*_) and/or S_4_(*B*_1*g*_), C_32_H_14_ appears to relax via internal conversion to S_2_(*B*_3*u*_), followed by fluorescence emission from S_2_(*B*_3*u*_). Further relaxation from S_2_(*B*_3*u*_) to S_1_(*B*_2*u*_) is not observed. The change in the emitting state from S_1_ to S_2_ with an increase in excitation energy reaching states S_2_ and S_3_/S_4_ indicates that internal conversion from S_2_(*B*_3*u*_) to S_1_(*B*_2*u*_) in C_32_H_14_ isolated in solid *para*-H_2_ is strongly suppressed, consistent with group theory, which states that direct transitions between these two states in the *D*_2*h*_ point group are parity and symmetry forbidden. Consequently, the S_2_(*B*_3*u*_) → S_0_(*A_g_*) emission was observed but mistakenly assigned to the S_1_(*B*_2*u*_) → S_0_(*A*_*g*_) emission, as quantum-chemical calculations and simulated spectra that could have pointed to the right direction were not available.

The most intense feature in the S_1_(*B*_2*u*_) ← S_0_(*A*_*g*_) excitation spectrum of C_32_H_14_ isolated in solid *para*-H_2_ is the 0_0_^0^ band located at ∼19 400 cm^–1^. We previously determined an average matrix (red) shift for vibronic transitions induced by the solid *para*-H_2_ environment of 70 ± 28 cm^–1^;^10^ consequently, we estimate that the S_1_(*B*_2*u*_)–S_0_(*A*_*g*_) origin band of C_32_H_14_ in the gaseous phase falls within the range of 512.9–514.4 nm. Recent DIB catalogues by Bondar^2^ and Fan et al.^3^ list DIBs at 513.7 nm and 513.04, 513.31, and 513.71 nm, respectively, close to our estimated S_1_(*B*_2*u*_)–S_0_(*A*_*g*_) 0_0_^0^ band position of C_32_H_14_ in the gaseous phase. These authors emphasize, however, that these DIBs are not certain due to their low intensity, blending with stellar lines, an “insufficient” number of detections, and/or an abnormal correlation to the degree of reddening (*E*_B–V_) of the observational sight lines considered. In addition to the S_1_(*B*_2*u*_)–S_0_(*A_g_*) 0_0_^0^ band, the fluorescence excitation spectrum of C_32_H_14_ isolated in solid *para*-H_2_ features another intense band at ∼435 nm (∼22 990 cm^–1^), which we previously assigned to the S_3_(*B*_1*g*_) or S_4_(*B*_1*g*_) state.^14^ In the gas-phase spectrum of C_32_H_14_, this feature should fall in the range of 433.2–434.2 nm. In this range, however, to the best of our knowledge, no DIB has been reported so far. According to the SpectroWeb 2.0 database^40–42^ and the Vienna Atomic Line Database (VALD3),^43–45^ an HI absorption band centered at 434.05 nm is commonly observed in the spectra of A, B, and O stars, which are common target stars in DIB surveys; identification of DIBs in this range in astronomical observations might therefore be challenging. As illustrated in Figure S3, vibronic features associated with the S_2_–S_0_ transition are weak compared to the S_1_–S_0_ origin band and the intense band in the S_3_/S_4_–S_0_ spectrum. As the DIB observed at ∼513 nm is designated as weak, the detection of DIBs corresponding to bands in the S_2_ ← S_0_ spectrum is rather unlikely. Indeed, to the best of our knowledge, no DIBs have been observed in the ranges of 454.3–455.4 and 439.1–440.2 nm, extrapolated from the two most intense peaks in the S_2_–S_0_ spectrum of C_32_H_14_ located at 456.3 and 441.0 nm, respectively, in solid *para*-H_2_. A contribution of C_32_H_14_ to the DIB spectrum, therefore, remains uncertain despite the possible coincidence of the S_1_(*B*_2*u*_)–S_0_(*A_g_*) 0_0_^0^ band with some DIBs based on the currently available data.

## Methods

The experiments and calculations presented here are similar to our previous work on C_32_H_14_ isolated in solid *para*-H_2_ ^14^ and follow the same methodology. Only a brief summary is provided here.

C_32_H_14_ and *para*-H_2_ were codeposited onto a nickel-coated copper plate at ∼3 K, which also served as a reflective surface for spectral measurements. Due to the low vapor pressure of C_32_H_14_, premixing with *para-*H_2_ was not feasible. Instead, a weak flow of *para*-H_2_ (flow rate of 13 *STP* L min^–1^, in which *STP* stands for the standard temperature of 273 K and the standard pressure of 760 Torr) was passed over a solid C_32_H_14_ sample heated to 160 °C. Matrix deposition typically lasted for 60–90 min. To monitor matrix composition, IR spectra were recorded with a Fourier-transform infrared (FTIR) spectrometer (Bruker, iFS66v) equipped with a KBr beam splitter and a Hg–Cd–Te detector cooled with liquid N_2_. Spectra were typically acquired over 300 scans, spanning the range of 500–5000 cm^–1^ at a resolution of 0.25 cm^–1^.

To record dispersed fluorescence spectra, the matrix was irradiated with the output of an optical parametric oscillator (OPO, EKSPLA NT340) pumped by an Nd:YAG laser (EKSPLA NT300), operated at 10 Hz. The laser beam was expanded to a diameter of ∼1.5 cm to maximize the overlap with the sample. Emitted light was collected with a convex lens (*f* = 50 mm), dispersed with a monochromator (Andor Shamrock SR500i, focal length of 0.5 m), and detected with an intensified charge-coupled device (iCCD, Andor iStar DH320T-18U-73, 1024 × 225 pixels, pixel size 26 μm × 26 μm). For emission measurements, a 1200 groove mm^–1^ grating blazed at 600 nm (reciprocal linear dispersion of 1.66 nm mm^–1^) was employed, and the width of the monochromator entrance slit was set to 25 μm. Under these conditions, each pixel of the iCCD corresponds to 0.043 nm, corresponding to <1.7 cm^–1^ in the range of 510–570 nm. Fluorescence emission was typically collected for 10 ns, starting 5 ns after excitation. All dispersed fluorescence spectra were corrected for wavelength-dependent variations in detector and grating sensitivity, and wavelengths were calibrated with a low-pressure Hg­(Ar) lamp.

Fluorescence excitation spectra were recorded by monitoring fluorescence emission within a fixed wavelength range while varying the OPO output wavelength in 0.1 nm increments. To enhance the fluorescence signal, a lower-resolution grating with 600 grooves mm^–1^ (500 nm blaze) was selected, and the monochromator slit width was increased to ∼2.5 mm. In the spectral range covered, the spectral line width of the OPO was ≤3 cm^–1^.

All quantum-chemical calculations were performed with the Gaussian 16 program package, revision A.03.^46^ Optimized geometries and harmonic vibrational frequencies of the electronic ground state and electronically excited singlet states were computed with the (TD-)­B3LYP-GD3BJ/6-311++G­(2d,2p) method. Based on the optimized geometries and scaled harmonic vibrational frequencies (scaling factor of 0.98)^14^ of the relevant electronic states, vibronic absorption and emission spectra were simulated using a Franck–Condon Herzberg–Teller approach. The computed stick spectra were convoluted with a Gaussian line shape with a fwhm of 20 or 55 cm^–1^ to facilitate direct comparison with the experimental data. No further corrections to model the matrix environment and its impact on the vibronic spectra have been applied.

## Supplementary Material





## Notes

Scaled harmonic vibrational wavenumbers of the ground and excited states of C_32_H_14_, simulated absorption and emission spectra for the three lowest-energy transitions of C_32_H_14_, fluorescence lifetime measurement, extended fluorescence excitation spectrum of C_32_H_14_ isolated in solid *para*-H_2_, and partial dispersed fluorescence spectra obtained with different excitation wavelengths (PDF)
Transparent Peer Review report available (PDF)
