# Supplementary material for: New Electronic Transition of Ovalene in Solid para-H2: The S1–S0 Transition with Its Origin at 19 400 cm–1
Source: J Phys Chem Lett. 2026 Jan 30;17(6):1735–41. doi: 10.1021/acs.jpclett.5c03826 (PMC12908154; doi:10.1021/acs.jpclett.5c03826)
Supplement: Supplementary file 1 [file jz5c03826_si_001.pdf]

Supporting Information

New Electronic Transition of Ovalene in Solid *Para*-H_2: the S_1–S_0 Transition with Origin at 19400 cm^{-1}

Isabelle Weber,[†] *Johanna Langner,*[†] *Henryk A. Witek,*[†] *and Yuan-Pern Lee*[†,‡]

[†] Department of Applied Chemistry and Institute of Molecular Science, National Yang Ming Chiao Tung University, Hsinchu 3000093, Taiwan

[‡] Center for Emergent Functional Matter Science, National Yang Ming Chiao Tung University, Hsinchu 300093, Taiwan

Emails: iweber@nycu.edu.tw (IW), yplee@nycu.edu.tw (YPL)

Table of Contents

Table S1. Scaled Harmonic Vibrational Wavenumbers of the Ground and the Six Lowest Excited States of $C_{32}H_{14}$ Calculated at the B3LYP-GD3BJ/6-311++G(2d,2p) Level of Theory S2

Figure S1. Comparison of simulated absorption (I) and emission (II) spectra of three lowest electronically excited singlet states S_1 (a), S_2 (b), and S_3 (c) of $C_{32}H_{14}$........ S5

Figure S2. Time-resolved integrated fluorescence (black symbols) upon excitation at 19444 cm^{-1} (514.3 nm) and a mono-exponential fit (red line) S6

Figure S3. Extended fluorescence excitation spectrum of $C_{32}H_{14}$ isolated in solid *para*-H_2 recorded by probing fluorescence emission in the range 17908−18235 cm^{-1} (558.4−566.4 nm) S7

Figure S4. Partial dispersed fluorescence spectra recorded upon excitation at three different wavelengths and comparison to the S_2 dispersed fluorescence spectrum of $C_{32}H_{14}$ isolated in solid *para*-H_2 .... S8

Table S1. Scaled Harmonic Vibrational Wavenumbers of the Ground and the Six Lowest Excited States of $C_{32}H_{14}$ Calculated at the B3LYP-GD3BJ/6-311++G(2d,2p) Level of Theory.[a]

mode	symmetry	S_0	S_1	S_2	S_3	S_4	S_5	S_6
v_1	a_g	3122	3123	3124	3126	3123	3126	3123
v_2	a_g	3119	3121	3121	3120	3121	3122	3119
v_3	a_g	3105	3106	3107	3108	3113	3107	3108
v_4	a_g	3101	3103	3103	3104	3104	3100	3103
v_5	a_g	1617	1603	1609	1592	1618	1590	1591
v_6	a_g	1604	1596	1579	1580	1596	1580	1573
v_7	a_g	1487	1501	1487	1480	1502	1479	1479
v_8	a_g	1468	1467	1461	1465	1484	1457	1452
v_9	a_g	1441	1422	1415	1420	1397	1424	1405
v_{10}	a_g	1366	1366	1368	1369	1388	1386	1363
v_{11}	a_g	1357	1356	1362	1355	1358	1361	1356
v_{12}	a_g	1316	1322	1323	1323	1322	1305	1319
v_{13}	a_g	1255	1244	1261	1241	1248	1236	1252
v_{14}	a_g	1215	1227	1215	1227	1238	1211	1210
v_{15}	a_g	1163	1157	1155	1151	1158	1146	1152
v_{16}	a_g	1144	1145	1133	1142	1148	1138	1134
v_{17}	a_g	1048	1049	1044	1047	1055	1033	1042
v_{18}	a_g	911	896	899	898	893	903	901
v_{19}	a_g	759	747	753	749	760	750	749
v_{20}	a_g	578	573	576	569	590	571	573
v_{21}	a_g	451	447	448	450	458	447	447
v_{22}	a_g	419	418	417	414	424	414	416
v_{23}	a_g	320	317	319	316	317	320	319
v_{24}	b_{1g}	3120	3121	3121	3124	3121	3129	3121
v_{25}	b_{1g}	3104	3106	3108	3109	3106	3107	3105
v_{26}	b_{1g}	3102	3103	3104	3103	3103	3105	3103
v_{27}	b_{1g}	1621	1565	1628	1557	1570	2300	1561
v_{28}	b_{1g}	1599	1547	1565	1500	1487	1563	1493
v_{29}	b_{1g}	1518	1499	1498	1473	1475	1496	1475
v_{30}	b_{1g}	1510	1469	1491	1422	1424	1457	1452
v_{31}	b_{1g}	1423	1418	1434	1396	1412	1423	1419
v_{32}	b_{1g}	1414	1393	1420	1392	1398	1376	1395
v_{33}	b_{1g}	1381	1363	1374	1312	1322	1349	1337
v_{34}	b_{1g}	1318	1321	1318	1225	1288	1262	1297
v_{35}	b_{1g}	1227	1213	1226	1196	1210	1218	1224
v_{36}	b_{1g}	1199	1191	1203	1157	1189	1171	1173
v_{37}	b_{1g}	1153	1130	1151	1121	1128	1125	1120
v_{38}	b_{1g}	1114	1114	1117	1059	1110	1101	1112
v_{39}	b_{1g}	891	893	884	867	884	899	878
v_{40}	b_{1g}	875	865	876	855	850	839	865
v_{41}	b_{1g}	706	707	706	699	705	704	703
v_{42}	b_{1g}	642	642	637	626	636	639	635

ν_{43}	b_{1g}	539	535	533	535	537	530	530
ν_{44}	b_{1g}	475	470	475	464	467	470	471
ν_{45}	b_{1g}	313	309	314	309	289	293	296
ν_{46}	b_{2g}	966	940	947	912	938	933	942
ν_{47}	b_{2g}	847	827	829	832	834	832	828
ν_{48}	b_{2g}	789	777	771	763	778	767	766
ν_{49}	b_{2g}	744	726	729	702	725	720	726
ν_{50}	b_{2g}	567	559	559	568	559	579	556
ν_{51}	b_{2g}	522	500	503	449	488	494	499
ν_{52}	b_{2g}	321	313	310	312	318	326	306
ν_{53}	b_{2g}	189	187	185	171	187	178	184
ν_{54}	b_{2g}	123	116	117	120	116	128	116
ν_{55}	b_{3g}	971	958	951	966	956	946	947
ν_{56}	b_{3g}	952	937	919	919	933	934	920
ν_{57}	b_{3g}	888	865	865	887	842	874	854
ν_{58}	b_{3g}	806	788	790	786	782	787	785
ν_{59}	b_{3g}	773	756	758	748	754	741	751
ν_{60}	b_{3g}	713	695	698	688	700	676	689
ν_{61}	b_{3g}	624	602	614	610	592	570	603
ν_{62}	b_{3g}	556	552	535	555	546	504	532
ν_{63}	b_{3g}	500	472	469	484	472	450	463
ν_{64}	b_{3g}	338	321	320	338	323	329	315
ν_{65}	b_{3g}	265	253	255	249	250	254	256
ν_{66}	b_{3g}	149	147	144	153	143	142	142
ν_{67}	a_{u}	970	958	949	966	956	945	945
ν_{68}	a_{u}	951	935	918	907	931	929	919
ν_{69}	a_{u}	819	808	804	794	814	800	799
ν_{70}	a_{u}	799	787	785	768	790	762	779
ν_{71}	a_{u}	657	653	641	643	650	647	636
ν_{72}	a_{u}	611	597	594	583	567	573	590
ν_{73}	a_{u}	471	463	446	450	469	461	441
ν_{74}	a_{u}	328	325	320	313	325	311	317
ν_{75}	a_{u}	252	245	248	248	240	247	244
ν_{76}	a_{u}	59	58	57	62	56	57	55
ν_{77}	b_{1u}	968	943	949	921	939	938	944
ν_{78}	b_{1u}	891	870	869	893	851	878	860
ν_{79}	b_{1u}	839	815	818	827	806	825	816
ν_{80}	b_{1u}	789	771	772	757	764	766	765
ν_{81}	b_{1u}	760	746	746	733	746	737	742
ν_{82}	b_{1u}	634	615	622	620	607	620	619
ν_{83}	b_{1u}	542	526	527	519	514	512	523
ν_{84}	b_{1u}	427	403	412	420	395	408	402
ν_{85}	b_{1u}	335	323	320	319	312	313	318
ν_{86}	b_{1u}	208	199	196	212	196	212	194
ν_{87}	b_{1u}	102	99	102	94	100	92	102
ν_{88}	b_{1u}	60	58	58	57	57	60	58

ν_{89}	b_{2u}	3120	3122	3122	3125	3121	3125	3122
ν_{90}	b_{2u}	3105	3107	3108	3109	3112	3107	3108
ν_{91}	b_{2u}	3103	3104	3106	3104	3106	3106	3104
ν_{92}	b_{2u}	3101	3102	3103	3103	3103	3100	3102
ν_{93}	b_{2u}	1630	1593	1576	1619	1564	1565	1731
ν_{94}	b_{2u}	1565	1542	1521	1542	1528	1538	1521
ν_{95}	b_{2u}	1533	1507	1507	1510	1498	1521	1517
ν_{96}	b_{2u}	1454	1435	1447	1438	1441	1445	1447
ν_{97}	b_{2u}	1446	1425	1423	1430	1385	1432	1442
ν_{98}	b_{2u}	1400	1328	1347	1402	1384	1359	1375
ν_{99}	b_{2u}	1313	1293	1300	1300	1292	1295	1301
ν_{100}	b_{2u}	1274	1265	1254	1271	1268	1251	1265
ν_{101}	b_{2u}	1227	1222	1222	1219	1220	1215	1215
ν_{102}	b_{2u}	1163	1149	1152	1141	1149	1149	1149
ν_{103}	b_{2u}	1073	1054	1062	1065	1060	1042	1066
ν_{104}	b_{2u}	918	916	910	917	915	917	909
ν_{105}	b_{2u}	786	779	764	786	774	785	770
ν_{106}	b_{2u}	707	704	701	704	701	703	698
ν_{107}	b_{2u}	657	654	653	653	656	651	653
ν_{108}	b_{2u}	577	573	572	579	570	574	581
ν_{109}	b_{2u}	492	483	481	491	477	489	493
ν_{110}	b_{2u}	276	273	275	272	271	275	274
ν_{111}	b_{3u}	3121	3123	3123	3125	3123	3126	3122
ν_{112}	b_{3u}	3119	3120	3120	3120	3120	3122	3121
ν_{113}	b_{3u}	3103	3104	3104	3108	3104	3106	3105
ν_{114}	b_{3u}	1615	1591	1558	1584	1600	1572	1718
ν_{115}	b_{3u}	1584	1552	1550	1551	1551	1550	1542
ν_{116}	b_{3u}	1495	1470	1455	1493	1486	1518	1526
ν_{117}	b_{3u}	1439	1439	1435	1441	1452	1445	1431
ν_{118}	b_{3u}	1412	1417	1403	1412	1405	1397	1393
ν_{119}	b_{3u}	1382	1374	1373	1387	1377	1380	1369
ν_{120}	b_{3u}	1380	1364	1330	1364	1364	1359	1356
ν_{121}	b_{3u}	1315	1288	1319	1350	1345	1340	1312
ν_{122}	b_{3u}	1242	1228	1237	1224	1229	1229	1234
ν_{123}	b_{3u}	1208	1218	1208	1202	1209	1206	1197
ν_{124}	b_{3u}	1170	1176	1176	1177	1178	1179	1157
ν_{125}	b_{3u}	1135	1143	1131	1136	1148	1139	1134
ν_{126}	b_{3u}	1071	1067	1067	1063	1064	1066	1057
ν_{127}	b_{3u}	978	972	969	974	972	978	962
ν_{128}	b_{3u}	780	769	775	768	773	770	773
ν_{129}	b_{3u}	677	670	669	668	657	671	670
ν_{130}	b_{3u}	537	535	535	532	534	531	539
ν_{131}	b_{3u}	425	417	419	422	419	406	435
ν_{132}	b_{3u}	392	390	388	393	393	388	386

[a]in cm^{-1}. Scaling factor is 0.98.

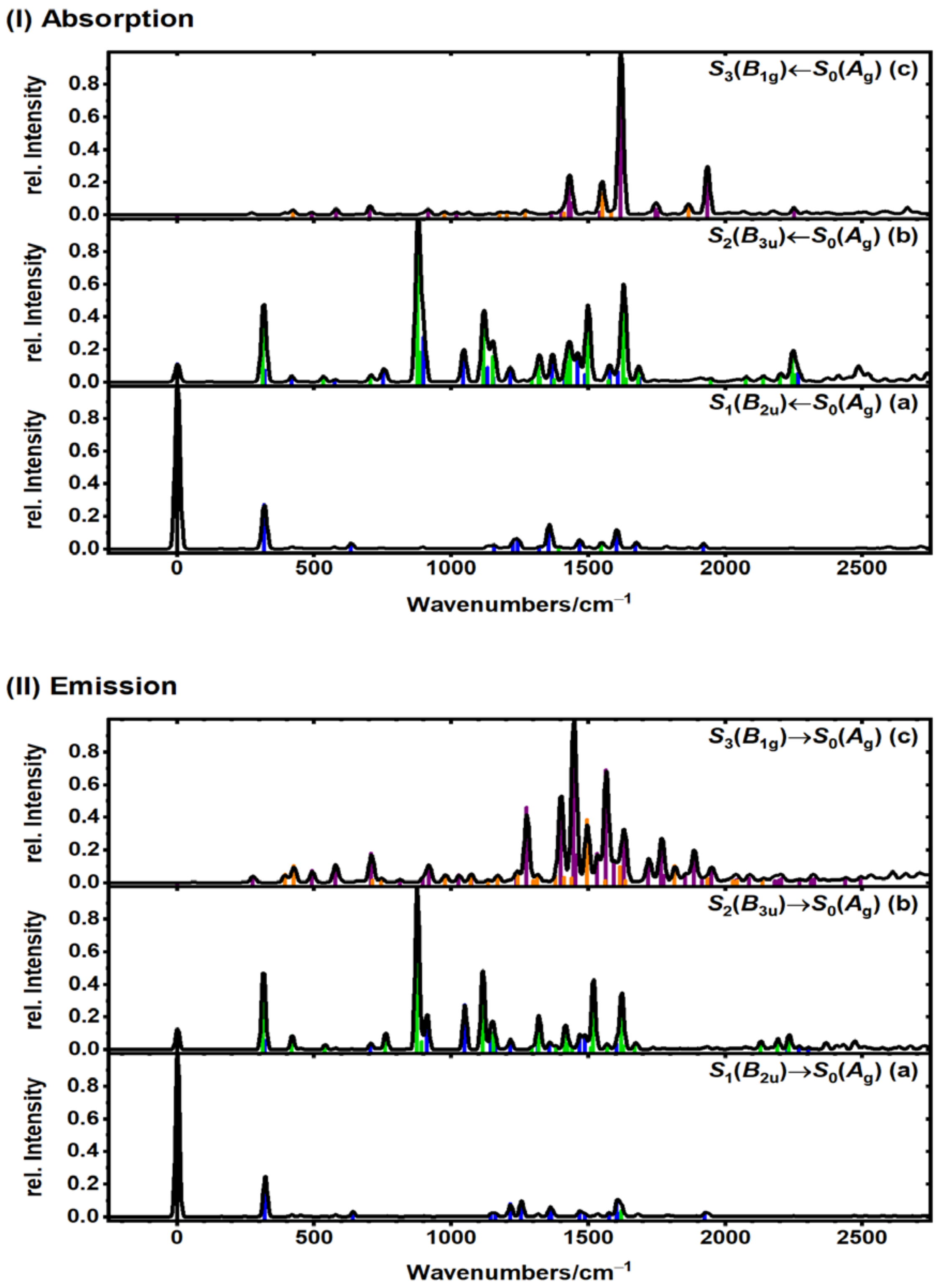

Figure S1. Comparison of simulated absorption (I) and emission (II) spectra of three lowest electronically excited singlet states S_1 (a), S_2 (b), and S_3 (c) of $C_{32}H_{14}$. Stick spectra were computed by a Franck-Condon Herzberg-Teller approach at the (TD-)B3LYP-GD3BJ/6-311++G(2d,2p) level of theory and convoluted with a Gaussian line shape with FWHM 20 cm^{-1}. Stick colors represent the symmetry of vibrational normal modes: a_g blue, b_{1g} green, b_{2u} violet, b_{3u} orange.

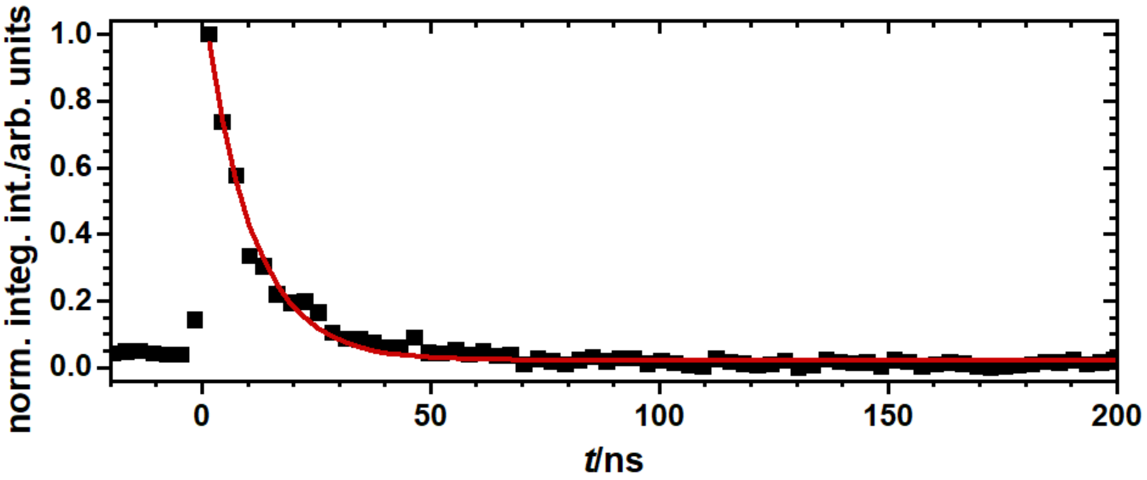

Figure S2. Time-resolved integrated fluorescence (black symbols) upon excitation at 19444 cm^{-1} (514.3 nm) and a mono-exponential fit (red line). Emission was detected for 3 ns every 3 ns and probed in the range 17636−17953 cm^{-1} (557.0−567.0 nm). From the fit $y = y_0 \times A \exp(-t/\tau)$, an emission lifetime of $\tau = 10 \pm 1$ ns was determined; the error corresponds to one standard deviation in fitting.

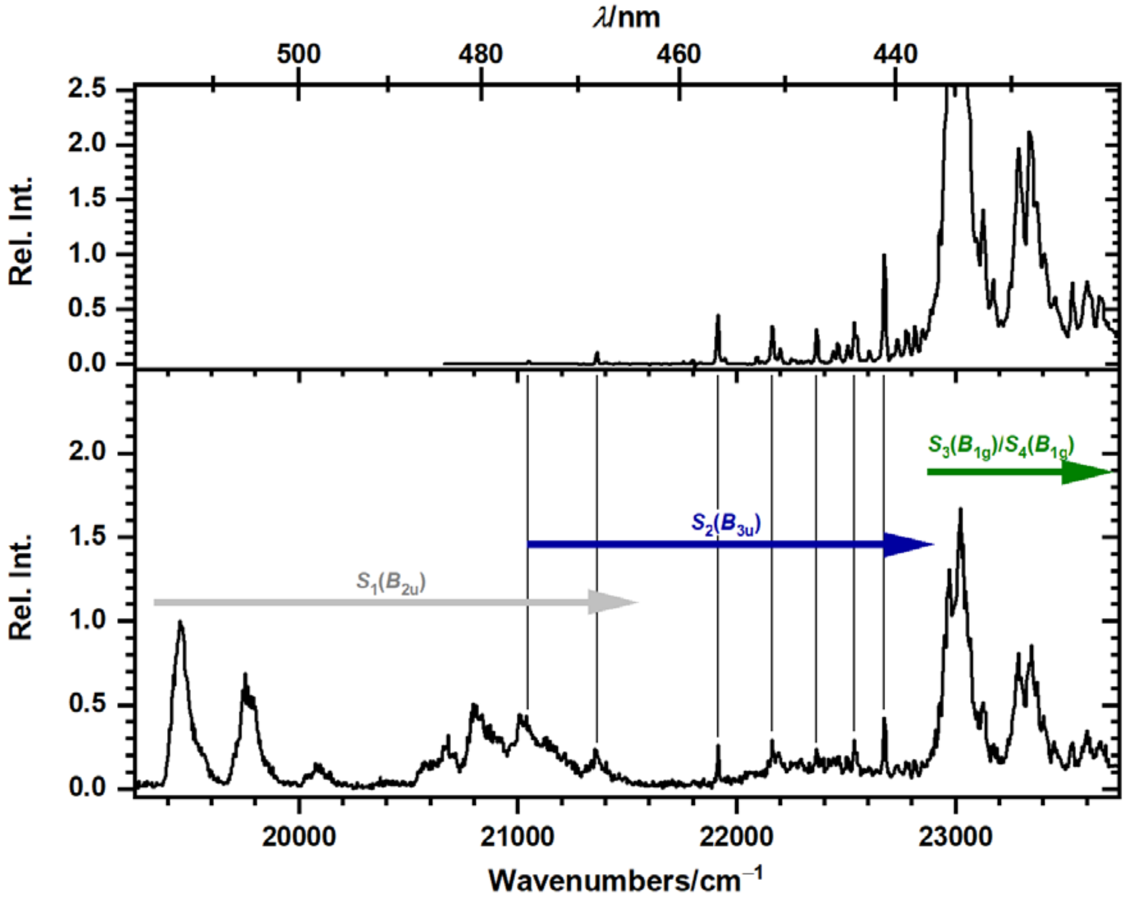

Figure S3. Extended fluorescence excitation spectrum of $C_{32}H_{14}$ isolated in solid *para*-H_2 recorded by probing fluorescence emission in the range 17908−18235 cm^{-1} (558.4−566.4 nm). The spectrum is normalized to the origin band of the $S_1(B_{2u})$−$S_0(A_g)$ transition. Assignments of various transitions are indicated. The previously reported $S_2(B_{3u})$←$S_0(A_g)$ fluorescence excitation spectrum of $C_{32}H_{14}$ isolated in solid *para*-H_2 obtained by probing emission from S_2 is depicted in the upper panel for comparison (originally published in Weber et al. *J. Chem. Phys. Lett.* **2024**, 15, 10696. licensed under CC BY 4.0).

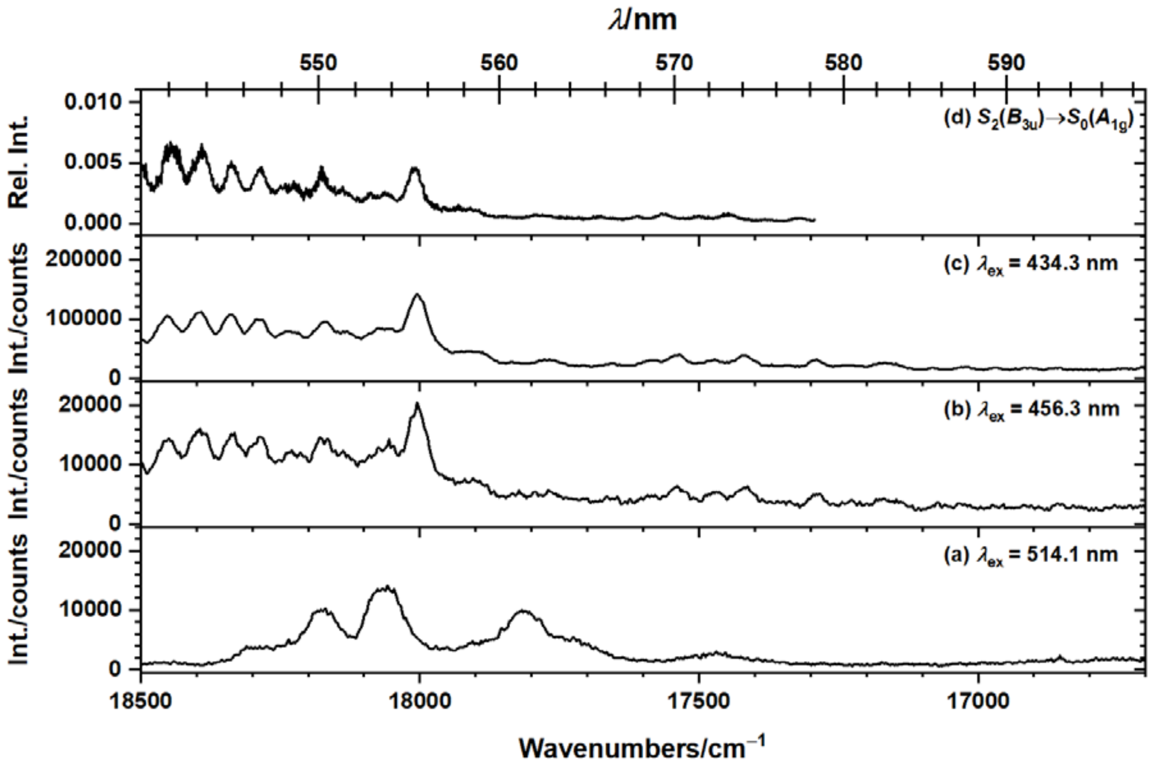

Figure S4. Partial dispersed fluorescence spectra recorded upon excitation at three different wavelengths and comparison to the reported S_2 dispersed fluorescence spectrum of $C_{32}H_{14}$ isolated in solid *para*-H_2. (a) Excitation at 514.1 nm, corresponding to excitation to the S_1 state. (b) Excitation at 453.6 nm, corresponding to excitation to the S_2 state. (c) Excitation at 434.3 nm, corresponding to excitation to a higher singlet state, most likely S_3 and/or S_4. (d) Previously reported $S_2(B_{3u})\rightarrow S_0(A_g)$ dispersed fluorescence spectrum of $C_{32}H_{14}$ isolated in solid *para*-H_2. The spectrum was recorded upon excitation at 431.1 nm and is normalised to the most intense peak in the overall spectrum at 20167 cm^{-1}, which is 883 cm^{-1} from the origin band, not shown (originally published in Weber et al. *J. Chem. Phys. Lett.* **2024**, 15, 10696. licensed under CC BY 4.0).
