# Supplementary material for: New Electronic Transition of Ovalene in Solid para-H2: The S1–S0 Transition with Its Origin at 19 400 cm–1
Source: J Phys Chem Lett. 2026 Jan 30;17(6):1735–41. doi: 10.1021/acs.jpclett.5c03826 (PMC12908154; doi:10.1021/acs.jpclett.5c03826)
Supplement: Supplementary file 2 [file jz5c03826_si_002.pdf]

jz-2025-03826d.R1

Name: Peer Review Information for "New Electronic Transition of Ovalene in Solid <i>Para</i>-H₂: the <i>S</i>₁–<i>S</i>₀ Transition with Origin at 19400 cm^{−1}"

First Round of Reviewer Comments

Reviewer: 1

Comments to the Author

Peer Review Report for Manuscript Submitted to The Journal of Physical Chemistry Letters

This manuscript describes a detailed experimental mapping of the low-lying electronic states of the overline molecule by observing in a *para* hydrogen matrix its emission spectrum following excitation at wavelengths of 480.6 and 431.1 nanometers. The excitation spectra were obtained by scanning the excitation laser wavelength while monitoring the emission at various wavelengths. The experimental work is complemented extremely well by quantum chemistry calculations.

The results of this work provide a precise determination of the electronic origin frequencies and their lifetimes for both the S_1 and S_2 states, as well as several vibrational frequencies for each of these states. As the authors point out, overline is an important PAH molecule, possibly associated with the diffuse interstellar bands. Using a weakly perturbing parahydrogen matrix has exciting potential for obtaining laboratory spectra to compare to the positions of known diffuse interstellar bands.

I could find no errors in the experimental work and highly recommend this manuscript for publication. My only suggestion is to improve the Table of Contents (TOC) graphic. Only after

studying Figure 1 extensively could I appreciate the existing graphic in the manuscript. Perhaps if the authors labeled the S_1 and $S_2 \rightarrow S_0$ emissions by their excitation wavelengths, it would make the TOC graphic more understandable.

Reviewer: 2

Comments to the Author

Journal: The *Journal of Physical Chemistry Letters*
Manuscript ID: jz-2025-03826d
Manuscript Type: Letter
Title: New Electronic Transition of Ovalene in Solid *Para*-H_2: the S_1–S_0 Transition with Origin at 19400 cm^{-1}

Complete List of Authors: Weber, Isabelle; National Yang Ming Chiao Tung University, Department of

Applied Chemistry and Institute of Molecular Science Langner, Johanna; National Yang Ming Chiao Tung

University, Department of Applied Chemistry and Institute of Molecular Science Witek, Henryk A.;

National Yang Ming Chiao Tung University, Department of Applied Chemistry and Institute of Molecular

Science Lee, Yuan-Pern; National Yang Ming Chiao Tung University, Department of Applied Chemistry and

Institute of Molecular Science; National Yang Ming Chiao Tung University, Center for Emergent Functional Matter Science

This letter reports a new set of electronic spectral features of ovalene ($C_{32}H_{14}$) approximately 1650 cm^{-1} below the previously reported S_2-S_0 origin that is assigned to the S_1-S_0 electronic transition based on comparison with quantum calculations. The identification of this new electronic transition provides further support for their previous re-assignment of the S_2-S_0 band that had mistakenly been assigned to the S_1-S_0 band. The major advance reported therefore is to clean up the electronic spectroscopy of ovalene in the 450 – 570 nm region where the highest density of vibronic absorption bands is observed

in the diffuse interstellar bands (DIB). These authors use para-hydrogen matrix isolation electronic spectroscopy instead of the more commonly employed jet-expansion and cryogenic ion trap methods, and thus this also reports the advantages (and disadvantages) of this somewhat novel technique over existing methods. The immediate significance of this advance is that it should provide more accurate laboratory spectra for this planar *peri*-condensed polycyclic aromatic hydrocarbon for comparison with astronomical observations in the DIB. However, in their previous publication they stated that, “A contribution of $C_{32}H_{14}$ to the DIB is therefore unlikely, in line with the conclusions previously drawn by Ehrenfreund et al. and Ruiterkamp et al.” In the current letter, while the DIB assignments are listed as motivation, no comment is made on whether the newly assigned features have any relevance to DIB features. Without this connection, the significance of this advance is the new assignment of an electronic transition in ovalene, but if a connection can be made this significantly increases the potential impact. The letter is well written and organized, and I only have a few comments related to clarity issues.

Comments

1. Do the new assignments provide insight into any of the features in the DIB?
2. In the abstract of ref. 18 they state, “The first spin-allowed $^1S_0(^1A_{1g})\rightarrow{}^1S_1(^1B_{3u}^-)$ transition with an origin at 21449 cm^{-1}, exhibits ...”. This confused me, is the reported origin band in solid parahydrogen at 19400 cm^{-1} or is it red shifted due to matrix shifts? Given that you are using pH_2 matrix isolation spectroscopy to assign low temperature gas phase transitions in the DIB, does the reported origin band reflect this, or are you estimating the gas phase values? This needs to be extremely clear.
3. Can you articulate simply why you were able to identify and assign the S_1-S_0 transition while others failed.

Reviewer: 3

Comments to the Author

Comments on jz-2025-03826d

The authors report dispersed fluorescence and fluorescence excitation spectra of ovalene (C32H14) isolated in solid para-H2. A band previously observed near 21050 cm⁻1 in a para-H2 matrix is reassigned to the S2–S0 origin transition. By extending the measurements to

lower energies, an additional band system with an origin at ~19400 cm^{-1} is newly observed and assigned to the S1–S0 transition. This assignment is supported by fluorescence lifetime measurements, TD-B3LYP-D3BJ/6-311++G(2d,2p) calculations, and Franck–Condon/Herzberg–Teller simulations.

Q1) Novelty relative to prior work

The authors previously reassigned the S1–S0 transition of ovalene reported in the gas-phase study by Amirav et al. (Ref. 18) to the S2–S0 transition using a para-H2 matrix isolation technique (Ref. 14). In the present manuscript, the same experimental approach is employed to extend the spectral window to lower energies, leading to the observation of a new band at ~19400 cm^{-1}, which is assigned to the S1 origin band.

However, aside from the experimental detection of this lower-energy origin band and a limited number of associated vibronic features, most of the electronic-structure information discussed in the manuscript—such as excitation energies of the six lowest singlet states, oscillator strengths, and excited-state lifetimes—appears to have already been reported in the previous gas-phase study (Ref. 18). Given that essentially the same spectroscopic methodology is used, it is not sufficiently clear what fundamentally new physical insight is gained beyond confirming the presence of the S1 spectrum under para-H2 matrix conditions.

The authors should therefore clarify more explicitly what new conclusions or conceptual advances are enabled by the present work in comparison with Ref. 18. As it currently stands, the manuscript appears to differ mainly in that the S1 spectrum is measured using the same para-H_2 matrix technique, without a clearly articulated advance beyond the earlier study.

Q2) Significance in the context of PAHs and diffuse interstellar bands

In the second paragraph on page 3, the authors emphasize that large PAHs—particularly their cationic and protonated derivatives—are considered promising candidates for carriers of diffuse interstellar bands (DIB), while also noting the experimental difficulty of obtaining gas-phase spectra under interstellar-medium-like conditions due to their low vapor pressures. Matrix isolation spectroscopy is then introduced as a strategy to overcome these limitations.

In this context, however, the present study focuses exclusively on electronically excited states of neutral ovalene rather than on ionic or protonated species. The authors should

therefore clarify the physical and astrochemical significance of identifying the previously unobserved S1–S0 transition of neutral ovalene. In particular, it would be helpful to explain how this result advances our understanding of the electronic structure and excited-state dynamics of large PAHs, and in what way such insight is relevant to ongoing efforts to identify or model DIB carriers.

As currently written, the connection between the DIB-motivated discussion in the Introduction and the neutral-state spectroscopy presented in the manuscript is not sufficiently articulated, and the broader significance of the reported S1–S0 assignment remains unclear.

Q3) Technical comments and questions

1. The fluorescence lifetimes reported in this work constitute one of the key arguments supporting the assignment of the S1 and S2 states. Measurements performed in solid para-H2 yield markedly different lifetimes for the two states, approximately ~1.7 μs for S_2 and ~10 ns for S1. While this contrast is striking, it is not obvious that these lifetimes can be regarded as intrinsic properties of gas-phase ovalene.

Although para-H2 is described as a soft matrix that interacts only weakly with guest molecules (page 3, bottom), it remains a condensed-phase environment. The authors should discuss whether matrix–guest interactions could differentially affect the nonradiative decay channels or radiative rates of the S1 and S2 states, and to what extent the observed lifetimes can be extrapolated to isolated, gas-phase molecules.

2. The reassignment of the 21449 cm^{-}1 band from S1 to S2 relies heavily on TD-DFT calculations combined with Franck–Condon/Herzberg–Teller simulations, fluorescence lifetimes, and oscillator-strength arguments. This interpretation therefore depends critically on the reliability of the TD-B3LYP-D3BJ/6-311++G(2d,2p) level of theory.

The authors should comment on the robustness of this assignment with respect to the choice of electronic-structure method. Are similar excitation energies, state orderings, and oscillator-strength patterns obtained with other TD-DFT functionals or with higher-level ab initio approaches?

3. The authors note that the agreement between experiment and simulation is less satisfactory for the excitation spectrum than for the emission spectrum. In particular, the

mean deviation in peak positions is reported as 11 ± 7 cm¯1 for emission, whereas it increases to 23 ± 17 cm¯1 for excitation.

The manuscript would benefit from a more detailed discussion of the origin of this discrepancy, including whether it arises primarily from experimental factors (e.g., spectral congestion or overlapping vibronic bands), limitations of the harmonic approximation, or matrix-induced perturbations that affect absorption and emission differently.

4. In the Franck–Condon/Herzberg–Teller simulations used to interpret the fluorescence spectra, it is unclear how the influence of the para-H_2 matrix is treated. The authors should clarify whether matrix effects are entirely neglected, implicitly incorporated through empirical line broadening, or considered in some other approximate manner.

5. Different Gaussian FWHM values (55 cm¯1 for one electronic state and 20 cm¯1 for the other) are used to reproduce the experimental spectra. While lifetime broadening may contribute to this difference, other effects—such as vibronic coupling strength, internal conversion efficiency, or site inhomogeneity—may also play a role. The physical basis for the chosen linewidths should be discussed more explicitly.

6. On page 4, lines 33–34, the value “21499 cm¯1” should be corrected to “21449 cm¯1”.

The manuscript reports an extension of previous spectroscopic studies of ovalene in solid para-H_2 to a lower-energy spectral region, leading to the observation of an additional band system and a revised assignment of the S1–S0 transition. The experimental work appears careful, and the interpretation is internally consistent. In this sense, the study has sufficient scientific merit and provides a useful refinement of the electronic-state assignments of ovalene.

Nevertheless, the advance reported here is largely incremental in nature. The primary contribution lies in expanding the measured spectral window and revising the interpretation accordingly, rather than in introducing a fundamentally new concept, methodology, or broadly impactful insight. When considered in the context of the authors’ own recent publications and the existing literature, the urgency and conceptual novelty required for The Journal of Physical Chemistry Letters are not fully evident.

Given the solid technical quality of the work and its value as a careful verification and refinement of earlier assignments, the manuscript may be more appropriately suited for a journal such as The Journal of Physical Chemistry A, where a detailed and thoroughly validated spectroscopic study of this kind would likely find a more natural and receptive audience.

Author's Response to Peer Review Comments:

We have revised the manuscript according to the reveiwers' comments. Please see the attached file for details.

We thank the reviewers for their valuable comments and suggestions. Below our detailed responses to the reviewer's comments. The reviewers' comments are in black, our responses and corrections in blue font. Revisions to the manuscript and electronic supplementary information are highlighted in blue.

Reviewer 1:

This letter reports a new set of electronic spectral features of ovalene ($C_{32}H_{14}$) approximately 1650 cm^{-1} below the previously reported S_2-S_0 origin that is assigned to the S_1-S_0 electronic transition based on comparison with quantum calculations. The identification of this new electronic transition provides further support for their previous re-assignment of the S_2-S_0 band that had mistakenly been assigned to the S_1-S_0 band. The major advance reported therefore is to clean up the electronic spectroscopy of ovalene in the 450 – 570 nm region where the highest density of vibronic absorption bands is observed in the diffuse interstellar bands (DIB). These authors use para-hydrogen matrix isolation electronic spectroscopy instead of the more commonly employed jet-expansion and cryogenic ion trap methods, and thus this also reports the advantages (and disadvantages) of this somewhat novel technique over existing methods. The immediate significance of this advance is that it should provide more accurate laboratory spectra for this planar *peri*-condensed polycyclic aromatic hydrocarbon for comparison with astronomical observations in the DIB. However, in their previous publication they stated that, "A contribution of $C_{32}H_{14}$ to the DIB is therefore unlikely, in line with the conclusions previously drawn by Ehrenfreund et al. and Ruiterkamp et al." In the current letter, while the DIB assignments are listed as motivation, no comment is made on whether the newly assigned features have any relevance to DIB features. Without this connection, the significance of this advance is the new assignment of an electronic transition in ovalene, but if a connection can be made this significantly increases the potential impact. The letter is well written and organized, and I only have a few comments related to clarity issues.

Comments

1. Do the new assignments provide insight into any of the features in the DIB?

The most intense feature in the reported S_1−S_0 fluorescence excitation spectrum of $C_{32}H_{14}$ is the 0_0^0 band located at ~19400 cm^{-1} in solid *para*-H_2. Adopting our previously determined matrix red shift of 70±28 cm^{-1} for solid *para*-H_2, the S_1−S_0 0_0^0 band of $C_{32}H_{14}$ in the gaseous phase is expected to be located at 512.9−514.4 nm. The DIB catalogue by Bondar (*Mont. Not. Roy. Astron. Soc.* **2012**, 423, 725.) lists a possible weak DIB at 513.70 nm. The Apache Point Observatory Catalogue of Fan et al. (*Astrophys. J.* **2019**, 878, 151.) includes three possible DIB at 513.036, 513.314, and 513.707 nm.

In addition to the S_1−S_0 origin band, the fluorescence excitation spectrum of $C_{32}H_{14}$ isolated in solid *para*-H_2 features an intense band ~435 nm, which we previously assigned to a higher electronically excited state (S_3 or S_4). In the gas-phase spectrum, this band is expected to fall in the range 433.2−434.2 nm. To the best of our knowledge, no DIB has been detected in this range so far, and the presence of a stellar atomic HI absorption line at 434.05 nm might hamper detection.

The most intense features in the $S_2 \leftarrow S_0$ spectrum of $C_{32}H_{14}$ are located at 456.3 and 441.0 nm in solid *para*-H_2, corresponding to 454.3−455.4 nm and 439.1−440.2 nm, respectively, in the gaseous phase. These bands, however, are weak compared to the $S_1 - S_0$ origin band and the intense band in the $S_3/S_4 \leftarrow S_0$ spectrum. As the DIB observed ~513 nm is designated as weak, detection of DIBs corresponding to bands in the $S_2 - S_0$ absorption spectrum of $C_{32}H_{14}$ is rather unlikely; indeed, to the best of our knowledge, no DIB has been observed in the ranges 454.3−455.4 nm and 439.1−440.2 nm so far.

No definitive conclusions about a possible contribution of $C_{32}H_{14}$ to the DIB can therefore be drawn: whilst the estimated $S_1 - S_0$ origin band position for $C_{32}H_{14}$ in the gaseous phase falls within close proximity of at least one possible DIB, no comparable agreement could be found for the intense feature previously assigned to a higher electronically excited singlet state. These conclusions, however, could not have been drawn without the observations presented in this manuscript.

We added the following paragraph on p.14f.. discussing a potential contribution of $C_{32}H_{14}$ to the DIB spectrum in view of the newly discovered transition: "The most intense feature in the $S_1(B_{2u}) \leftarrow S_0(A_g)$ excitation spectrum of $C_{32}H_{14}$ isolated in solid *para*-H_2 is the 0^0_0 band located at ~19400 cm^{-1}. We previously determined an average matrix (red) shift for vibronic transitions induced by the solid *para*-H_2 environment of 70 ± 28 cm^{-1};[10] consequently, we estimate that the $S_1(B_{2u}) - S_0(A_g)$ origin band of $C_{32}H_{14}$ in the gaseous phase falls within 512.9−514.4 nm. Recent DIB catalogues by Bondar[2] and Fan et al.[3] list DIB at 513.7 nm, and 513.04, 513.31, and 513.71 nm, respectively, close to our estimated $S_1(B_{2u}) - S_0(A_g)$ 0^0_0 band position of $C_{32}H_{14}$ in the gaseous phase. These authors emphasize, however, that these DIB are not certain due to low intensity, blending with stellar lines, an "insufficient" number of detections, and/or an abnormal correlation to the degree of reddening (E_{B-V}) of the observational sightlines considered. In addition to the $S_1(B_{2u}) - S_0(A_g)$ 0^0_0 band, the fluorescence excitation spectrum of $C_{32}H_{14}$ isolated in solid *para*-H_2 features another intense band at ~435 nm (~22990 cm^{-1}), which we previously assigned to the $S_3(B_{1g})$ or $S_4(B_{1g})$ state.[14] In the gas-phase spectrum of $C_{32}H_{14}$, this feature should fall in the range of 433.2−434.2 nm. In this range, however, to the best of our knowledge, no DIB has been reported so far. According to the SpectroWeb 2.0 database[36,37,38] and the Vienna Atomic Line Database (VALD3),[39,40,41] an HI absorption band centred at 343.04 nm is commonly observed in the spectra of A, B, and O stars, which are common target stars in DIB surveys; identification of DIB in this range in astronomical observations might therefore be challenging. As illustrated in Figure S3, vibronic features associated with the $S_2 - S_0$ transition are weak compared to the $S_1 - S_0$ origin band and the intense band in the $S_3/S_4 - S_0$ spectrum. As the DIB observed at ~513 nm is designated as weak, detection of DIBs corresponding to bands in the $S_2 \leftarrow S_0$ spectrum is rather unlikely. Indeed, to the best of our knowledge, no DIB have been observed in the ranges 454.3−455.4 and 439.1−440.2 nm, extrapolated from the two most intense peaks in the $S_2 - S_0$ spectrum of $C_{32}H_{14}$ located at 456.3 and 441.0 nm in solid *para*-H_2, respectively. A contribution of $C_{32}H_{14}$

to the DIB spectrum, therefore, remains uncertain despite the possible coincidence of $S_1(B_{2u})-S_0(A_g)$ 0_0^0 band with some DIB based on the currently available data."

2. In the abstract of ref. 18 they state, "The first spin-allowed $^1S_0(^1A_{1g})\rightarrow{}^1S_1(^1B_{3u}$-) transition with an origin at 21449 cm^{-1}, exhibits …". This confused me, is the reported origin band in solid parahydrogen at 19400 cm^{-1} or is it red shifted due to matrix shifts? Given that you are using pH_2 matrix isolation spectroscopy to assign low temperature gas phase transitions in the DIB, does the reported origin band reflect this, or are you estimating the gas phase values? This needs to be extremely clear.

We compared the gas-phase spectra of jet-cooled $C_{32}H_{14}$ published by Amirav et al. (ref. 18, *J. Chem. Phys.* **1981**, 74, 3756.) in detail with our *para*-H_2 matrix isolation spectra in our previous publication (Weber et al. *J. Phys. Chem. Lett.* **2024**, 15, 10696.) and might therefore not have been clear enough in the present manuscript. In short, in our 2024 publication, we proposed that the spectra reported by Amirav et al., with their origin band at 21449 cm^{-1}, were mistakenly assigned to the S_1–S_0 transition of $C_{32}H_{14}$ and should instead be assigned to the S_2–S_0 transition. We therefore refer to the S_1–S_0 transition reported by Amirav et al. as the S_2–S_0 transition in the present manuscript.

This is now explicitly stated on p. 5: " Consistent with our proposed reassignment, we will refer to the spectra published by Amirav et al.[18,19] as the $S_2(B_{3u})$–$S_0(A_g)$ spectra of jet-cooled $C_{32}H_{14}$, rather than the $S_1(B_{2u})$–$S_0(A_g)$ spectra as in the original publications from 1980 and 1981."

In general, peak positions reported in this manuscript and the preceding one (Weber et al. *J. Phys. Chem. Lett.* **2024**, 15, 10696.) refer to peak positions in solid *para*-H_2 and, unless explicitly stated, have not been corrected for the influence of the *para*-H_2 environment. Only when we compared our experimental data to astronomical observations, i.e. DIB positions, did we apply an empirical correction for the matrix shift. We now clarify peak positions in solid *para*-H_2 in the abstract ("[…] origin band at ~19400 cm^{-1} in solid *para*-H_2 […]"), on p. 4 ("[…] a weak origin band at 21050 ± 3 cm^{-1} in solid *para*-H_2 […]"), on p.8 ("[…] at 19400 ± 8 cm^{-1} in solid *para*-H_2, […]"), on p.12 ("[…] at 19400 ± 8 cm^{-1} in solid *para*-H_2 under identical experimental conditions reinforces our previous reassignment of the gaseous spectrum of the $S_1(B_{2u})$–$S_0(A_g)$ transition to the $S_2(B_{3u})$–$S_0(A_g)$ transition of $C_{32}H_{14}$, with an origin band at 21050 ± 3 cm^{-1} in solid *para*-H_2.)" and on p.13 ("[…] the range 19400–21500 cm^{-1} in solid *para*-H_2, […]").

3. Can you articulate simply why you were able to identify and assign the S_1-S_0 transition while others failed.

Fundamental to our identification and assignment of the S_1–S_0 transition was the combination of experiment and theory. Internal conversion between S_2 and S_1 does not occur in $C_{32}H_{14}$ isolated in solid *para*-H_2, and we expect it to be negligible also in the gaseous phase due to the low number of vibrational states of appropriate symmetry to couple the two electronic states. Therefore, upon excitation of $C_{32}H_{14}$ to S_2 (or a higher excited singlet state), only emission from S_2 is observed, and S_1 can be easily overlooked without the support of quantum-chemical calculations and simulated spectra. These were not available to Amirav et al. in 1980.

This aspect is now explicitly mentioned on p.14: "Consequently, the $S_2(B_{3u}) \rightarrow S_0(A_g)$ emission was observed but mistakenly assigned to the $S_1(B_{2u}) \rightarrow S_0(A_g)$ emission as quantum-chemical calculations and simulated spectra that could have pointed to the right direction were not available."

Reviewer 2:

This manuscript describes a detailed experimental mapping of the low-lying electronic states of the overline molecule by observing in a para hydrogen matrix its emission spectrum following excitation at wavelengths of 480.6 and 431.1 nanometers. The excitation spectra were obtained by scanning the excitation laser wavelength while monitoring the emission at various wavelengths. The experimental work is complemented extremely well by quantum chemistry calculations. The results of this work provide a precise determination of the electronic origin frequencies and their lifetimes for both the S_1 and S_2 states, as well as several vibrational frequencies for each of these states. As the authors point out, overline is an important PAH molecule, possibly associated with the diffuse interstellar bands. Using a weakly perturbing parahydrogen matrix has exciting potential for obtaining laboratory spectra to compare to the positions of known diffuse interstellar bands. I could find no errors in the experimental work and highly recommend this manuscript for publication. My only suggestion is to improve the Table of Contents (TOC) graphic. Only after studying Figure 1 extensively could I appreciate the existing graphic in the manuscript. Perhaps if the authors labeled the S_1 and $S_2 \rightarrow S_0$ emissions by their excitation wavelengths, it would make the TOC graphic more understandable.

Following the reviewers' suggestions, we have now additionally labelled the experimental traces in the TOC figure with the excitation wavelengths employed in their acquisition and the assignments for the corresponding electronically excited states.

Reviewer 3:

Recommendation: Reconsider as an article in The Journal of Physical Chemistry A/B/C.

Comments:

Comments on jz-2025-03826d

The authors report dispersed fluorescence and fluorescence excitation spectra of ovalene (C32H14) isolated in solid para-H2. A band previously observed near 21050 cm¯1 in a para-H2 matrix is reassigned to the S2–S0 origin transition. By extending the measurements to lower energies, an additional band system with an origin at ~19400 cm¯1 is newly observed and assigned to the S1–S0 transition. This assignment is supported by fluorescence lifetime measurements, TD-B3LYP-D3BJ/6-311++G(2d,2p) calculations, and Franck–Condon/ Herzberg–Teller simulations.

Q1) Novelty relative to prior work
The authors previously reassigned the S1–S0 transition of ovalene reported in the gas-phase study by Amirav et al. (Ref. 18) to the S2–S0 transition using a para-H2 matrix isolation technique (Ref. 14). In the present manuscript, the same experimental approach is employed to extend the spectral window to lower energies, leading to the observation of a new band at ~19400 cm¯1, which is assigned to the S1 origin band.

However, aside from the experimental detection of this lower-energy origin band and a limited number of associated vibronic features, most of the electronic-structure information discussed in the manuscript—such as excitation energies of the six lowest singlet states, oscillator strengths, and excited-state lifetimes—appears to have already been reported in the previous gas-phase study (Ref. 18). Given that essentially the same spectroscopic methodology is used, it is not sufficiently clear what fundamentally new physical insight is gained beyond confirming the presence of the S1 spectrum under para-H2 matrix conditions.

The authors should therefore clarify more explicitly what new conclusions or conceptual advances are enabled by the present work in comparison with Ref. 18. As it currently stands, the manuscript appears to differ mainly in that the S1 spectrum is measured using the same para-H_2 matrix technique, without a clearly articulated advance beyond the earlier study.

In comparison to reference 18 in which Amirav et al. present the (now) S_2–S_0 dispersed fluorescence and fluorescence excitation spectra of jet-cooled $C_{32}H_{14}$, the present manuscript reports the identification of a previously unobserved electronically excited state of $C_{32}H_{14}$, namely its S_1 state, and a detailed explanation of why this state might have been overlooked so far (c.f. our reply to Reviewer 1, Comment 3). The newly observed transition falls into a spectral range strongly relevant to the DIB problem; new insights with respect to the DIB are discussed in our reply to Reviewer 1, Comment 1).

It is true that the presented results have been obtained and analysed in a very similar way as our previously reported reassignment of the S_2 spectra of $C_{32}H_{14}$ (ref. 14). Our previous results inspired us to look for the missing S_1 state and we were successful in locating this S_1 electronic transition with a much shorter lifetime. Without the current results, however, the previously reported S_2 assignment ultimately remained questionable in view of the uncertainty of TD-DFT calculations with respect to the electronic excited state order of D_{2h} symmetric molecules. Beyond the presentation of a previously unobserved electronic excited state, we feel that the thorough comparison of experiment and theory will therefore be of interest for theoreticians as well.

Q2) Significance in the context of PAHs and diffuse interstellar bands

In the second paragraph on page 3, the authors emphasize that large PAHs—particularly their cationic and protonated derivatives—are considered promising candidates for carriers of diffuse interstellar bands (DIB), while also noting the experimental difficulty of obtaining gas-phase spectra under interstellar-medium-like conditions due to their low vapor pressures. Matrix isolation spectroscopy is then introduced as a strategy to overcome these limitations.

In this context, however, the present study focuses exclusively on electronically excited states of neutral ovalene rather than on ionic or protonated species. The authors should therefore clarify the physical and astrochemical significance of identifying the previously unobserved S1–S0 transition of neutral ovalene. In particular, it would be helpful to explain how this result advances our understanding of the electronic structure and excited-state dynamics of large PAHs, and in what way such insight is relevant to ongoing efforts to identify or model DIB carriers.

As currently written, the connection between the DIB-motivated discussion in the Introduction and the neutral-state spectroscopy presented in the manuscript is not sufficiently articulated, and the broader significance of the reported S1–S0 assignment remains unclear.

For a discussion of the presented results in relation to the DIB problem, please refer to our reply to Reviewer 1, Comment 1.

Q3) Technical comments and questions

1. The fluorescence lifetimes reported in this work constitute one of the key arguments supporting the assignment of the S1 and S2 states. Measurements performed in solid para-H2 yield markedly different lifetimes for the two states, approximately ~1.7 μs for S_2 and ~10 ns for S1. While this contrast is striking, it is not obvious that these lifetimes can be regarded as intrinsic properties of gas-phase ovalene.

Although para-H2 is described as a soft matrix that interacts only weakly with guest molecules (page 3, bottom), it remains a condensed-phase environment. The authors should discuss whether matrix–guest interactions could differentially affect the nonradiative decay channels or radiative rates of the S1 and S2 states, and to what extent the observed lifetimes can be extrapolated to isolated, gas-phase molecules.

We agree with the reviewer that the influence of the matrix host cannot be categorically ignored when comparing results obtained from matrix isolation and gas-phase experiments. For the S_1 state of coronene isolated in solid *para*-H_2, we determined a fluorescence lifetime of ~450 ns, consistent with lifetime of coronene in the gaseous phase reported by Kunishige et al. (382−452 ns, *J. Chem. Phys.* **2017**, 146, 044309.) and Bermudez et al. (354−550 ns, *J. Phys. Chem.* **1986**, 21, 5029.). For the S_2 state of $C_{32}H_{14}$, we inferred a fluorescence lifetime of ~1.7 μs in solid *para*-H_2 within the range of fluorescence lifetimes 1.7−2.4 μm reported by Amirav et al. for $C_{32}H_{14}$ in the gaseous phase.

Usually, in matrix environments, higher electronically excited states relax quickly to the lowest electronically excited state of the same spin multiplicity, and emission is only observed from this state. Consequently, emission lifetimes determined in matrix-isolation experiments typically do not vary significantly with excitation energy. Due to the suppressed S_2–S_1 intersystem crossing, this is not the case for $C_{32}H_{14}$, and we observed two significantly different lifetimes depending on the excited state probed. We expect a similar behaviour for $C_{32}H_{14}$ in the gaseous phase, as the density of vibrational states of suitable symmetry to couple S_1 and S_2 in the vicinity of the S_2 potential energy surface minimum is small.

We now comment on the influence of the *para*-H_2 matrix environment on fluorescence lifetimes on p.5: "[…]; the agreement of fluorescence lifetimes of jet-cooled $C_{32}H_{14}$ and $C_{32}H_{14}$ isolated in solid *para*-H_2 is consistent with our earlier experiments, e.g. on coronene ($C_{24}H_{12}$, $\tau_{fl}^{pH_2}$ ~450 ns, τ_{fl}^{jet}=354−550 ns),[20,21] suggesting small influence of the solid *para*-H_2 environment on fluorescence lifetimes."

2. The reassignment of the 21449 cm⁻1 band from S1 to S2 relies heavily on TD-DFT calculations combined with Franck–Condon/Herzberg–Teller simulations, fluorescence

lifetimes, and oscillator-strength arguments. This interpretation therefore depends critically on the reliability of the TD-B3LYP-D3BJ/6-311++G(2d,2p) level of theory.

The authors should comment on the robustness of this assignment with respect to the choice of electronic-structure method. Are similar excitation energies, state orderings, and oscillator-strength patterns obtained with other TD-DFT functionals or with higher-level ab initio approaches?

We share the reviewers' concerns regarding the robustness and accuracy of TD-DFT calculations. It has previously been demonstrated that TD-DFT wrongly predicts the order of the lowest excited singlet states for some PAH of D2h symmetry, such as naphthalene and pyrene. For larger acenes, however, state ordering obtained from TD-DFT calculations is consistent with higher-level *ab initio* calculations at the CC2 level.

Ayanpour et al. (*J. Chem. Phys.* **2014**, 140, 104301) computed one- and two-photon states of $C_{32}H_{14}$ at the PPP-MRSDCI level of theory. Their calculations predict symmetries of B_{2u} and B_{3u} for the S_1 and S_2 states, respectively, in agreement with our TD-DFT calculations. To the best of our knowledge, this is the only higher-level quantum-chemical study of $C_{32}H_{14}$ currently available in the literature

A comparison of the presented TD-B3LYP-GD3BJ/6-311++G(2d,2p) calculations with TD-B3PW91/6-311++G(2d,2p) calculations demonstrates high consistency between the two (TD-)DFT methods: they predict identical excited-state orders and comparable oscillator strengths. Simulated S_1–S_0 emission spectra obtained with the two methods are also consistent, although the relative intensities vary somewhat. It must be kept in mind that this comparison of two functionals cannot be extrapolated, and other DFT functionals might perform very differently.

Our experimental observation of the S_1 state further supports the the reliability of the TD-B3LYP-D3BJ/6-311++G(2d,2p) level of theory

We now comment on the reliability and robustness of our computational approach on p.5f. ("Benkyi et al.[22] previously demonstrated that TD-DFT failed to predict the correct order of the lowest excited singlet states S_1 and S_2 for naphthalene and pyrene, two molecules also belonging to the D_{2h} point group. To the best of our knowledge, only one higher-level quantum-chemical investigation of the electronically excited states of $C_{32}H_{14}$ has been published up to today. Employing the PPP-MRSDCI method, Ayanpour et al.[23] predict the two lowest excited singlet one-photon states to be of $B_{2u}(S_1)$ and $B_{3u}(S_2)$ symmetry, respectively, consistent with our TD-DFT results."). As our set of spectra was simulated using only different DFT methods and, in our opinion, therefore insufficient for generalization, we decided not to include the functional comparison in the manuscript to avoid confusion.

3. The authors note that the agreement between experiment and simulation is less satisfactory for the excitation spectrum than for the emission spectrum. In particular, the mean deviation in peak positions is reported as 11 ± 7 cm^{-1} for emission, whereas it increases to 23 ± 17 cm^{-1} for excitation.

The manuscript would benefit from a more detailed discussion of the origin of this discrepancy, including whether it arises primarily from experimental factors (e.g., spectral congestion or overlapping vibronic bands), limitations of the harmonic approximation, or matrix-induced perturbations that affect absorption and emission differently.

The larger mean deviation in peak positions between experiment and simulations for fluorescence excitation spectra compared to dispersed fluorescence spectra is consistent with our previous studies of PAH and PAH derivatives isolated in solid *para*-H_2 and likely related to our treatment of simulated spectra:

We have applied the same vibrational scaling factors to all simulated spectra −emission and absorption− although the former probe the ground state PES and the latter probe the excited state PES. Vibrational scaling factors were determined by comparison of computed harmonic vibrational wavenumbers (at the level of theory employed in this study) to vibrational wavenumbers determined from *para*-H_2 matrix isolation IR spectra and are therefore, strictly speaking, only applicable to the electronic ground state (dispersed fluorescence). As, to the best of our knowledge, no experimentally determined vibrational wavenumbers for electronically excited states of molecules isolated in solid *para*-H_2 are currently available, determining a second vibrational scaling factor for excited-state wavenumbers is impossible. We therefore decided to apply ground state vibrational scaling factors to all simulated spectra −emission and absorption− anticipating larger deviations in peak positions in the case of our fluorescence excitation spectra.

We now explicitly comment on the causes of deviations between experimental and simulated spectra on p.9 ("[…] likely because of the significant overlap of vibronic contributions >1000 cm^{-1}. Nonetheless, it […]") and p.11 ("[…] likely due to uncertainties in the experimental peak positions due to significant line broadening and the somewhat low S/N ratio. The larger deviations in peak positions for the fluorescence excitation spectrum, as compared to the dispersed fluorescence spectrum, however, are consistent with our previous work,[11,13,17] and likely associated with the differences in accuracy of (TD)-DFT for the description of ground and excited state potential energy surfaces and the questionable applicability of ground-state vibrational scaling factors to excited-state vibrational wavenumbers.")

4. In the Franck–Condon/Herzberg–Teller simulations used to interpret the fluorescence spectra, it is unclear how the influence of the para-H_2 matrix is treated. The authors should clarify whether matrix effects are entirely neglected, implicitly incorporated through empirical line broadening, or considered in some other approximate manner.

Matrix effects induced by the solid *para*-H_2 environment have not been explicitly considered in the presented simulations, except for the empirical scaling of harmonic vibrational wavenumbers.

We added the following sentence at the end of the method section on p.16.: "No further corrections to model the matrix environment and its impact on the vibronic spectra have been applied."

5. Different Gaussian FWHM values (55 cm¯1 for one electronic state and 20 cm¯1 for the other) are used to reproduce the experimental spectra. While lifetime broadening may

contribute to this difference, other effects—such as vibronic coupling strength, internal conversion efficiency, or site inhomogeneity—may also play a role. The physical basis for the chosen linewidths should be discussed more explicitly.

To facilitate comparison between experimental and computational spectra, we selected a Gaussian fwhm that best fits the experimental data. From a physical perspective, linewidths in matrix isolation spectra are influenced by the intrinsic properties of the observed electronically excited states/electronic transitions (lifetime, internal conversion efficiency, etc.), the matrix environment (differing substitution sites, coupling to lattice vibrations, etc.), and instrumental factors. From an excited state lifetime of ~10 ns (S_1 state of $C_{32}H_{14}$), a linewidth of ~0.0005 cm^{-1} can be estimated, accounting only for lifetime broadening; this is several orders of magnitude smaller than the experimentally observed fwhm of ~55 cm^{-1}. Likewise, the experimentally achievable resolution of a <3 cm^{-1} is not sufficient to explain the observed spectral linewidths, and it is therefore safe to assume that these mainly originate from the coupling of the electronic/vibrational energy levels of the isolated guest molecules with the matrix lattice vibrations.

We now comment on this on p.8: "The significantly different emission lifetimes, however, do not explain the notable differences in linewidth between the experimental spectra, c.f. Figure 1(a) and (c), as lifetime broadening can be estimated to be ~0.0005 cm^{-1} for the shorter-lived $S_1(B_{2u})$ state; instead, line broadening will be mainly related to the coupling of vibronic energy levels of the isolated guest molecules to lattice vibrations of the *para*-H_2 crystal."

6. On page 4, lines 33–34, the value "21499 cm¯1" should be corrected to "21449 cm¯1".

The corrected passage now reads "[…] at 21449 × 10 cm^{-1} […]".

The manuscript reports an extension of previous spectroscopic studies of ovalene in solid para-H_2 to a lower-energy spectral region, leading to the observation of an additional band system and a revised assignment of the S1–S0 transition. The experimental work appears careful, and the interpretation is internally consistent. In this sense, the study has sufficient scientific merit and provides a useful refinement of the electronic-state assignments of ovalene.

Nevertheless, the advance reported here is largely incremental in nature. The primary contribution lies in expanding the measured spectral window and revising the interpretation accordingly, rather than in introducing a fundamentally new concept, methodology, or broadly impactful insight. When considered in the context of the authors' own recent publications and the existing literature, the urgency and conceptual novelty required for The Journal of Physical Chemistry Letters are not fully evident.

Given the solid technical quality of the work and its value as a careful verification and refinement of earlier assignments, the manuscript may be more appropriately suited for a journal such as The Journal of Physical Chemistry A, where a detailed and thoroughly validated spectroscopic study of this kind would likely find a more natural and receptive audience.

Additional Questions:

Urgency: Moderate

Significance: Moderate

Novelty: Moderate

Scholarly Presentation: Moderate

Is the paper likely to interest a substantial number of physical chemists, not just specialists working in the authors' area of research?: No
